# Ephemeral data handling in microservices with Tquery

**DOI:** 10.7717/peerj-cs.1037

**Published:** 2022-07-22

**Authors:** Saverio Giallorenzo, Fabrizio Montesi, Larisa Safina, Stefano Pio Zingaro

**Affiliations:** 1 Università di Bologna, Bologna, Italy; 2 INRIA, Sophia Antipolis, France; 3 University of Southern Denmark, Odense, Denmark; 4 INRIA, Lille, France

**Keywords:** Microservices, Jolie, Semi-structured data, Ephemeral data, Edge computing, Fog computing, Formal methods, Service-oriented computing, Query languages, e-Health

## Abstract

The adoption of edge and fog systems, along with the introduction of privacy-preserving regulations, compel the usage of tools for expressing complex data queries in an ephemeral way. That is, queried data should not persist. Database engines partially address this need, as they provide domain-specific languages for querying data. Unfortunately, using a database in an ephemeral setting has inessential issues related to throughput bottlenecks, scalability, dependency management, and security (*e.g.*, query injection). Moreover, databases can impose specific data structures and data formats, which can hinder the development of microservice architectures that integrate heterogeneous systems and handle semi-structured data. In this article, we present Jolie/Tquery, the first query framework designed for ephemeral data handling in microservices. Jolie/Tquery joins the benefits of a technology-agnostic, microservice-oriented programming language, Jolie, and of one of the most widely-used query languages for semi-structured data in microservices, the MongoDB aggregation framework. To make Jolie/Tquery reliable for the users, we follow a cleanroom software engineering process. First, we define Tquery, a theory for querying semi-structured data compatible with Jolie and inspired by a consistent variant of the key operators of the MongoDB aggregation framework. Then, we describe how we implemented Jolie/Tquery following Tquery and how the Jolie type system naturally captures the syntax of Tquery and helps to preserve its invariants. To both illustrate Tquery and Jolie/Tquery, we present the use case of a medical algorithm and build our way to a microservice that implements it using Jolie/Tquery. Finally, we report microbenchmarks that validate the expectation that, in the ephemeral case, using Jolie/Tquery outperforms using an external database (MongoDB, specifically).

## Introduction

### Background

Modern applications that make use of Edge Computing (Shi et al., 2016) and the Internet of Things (IoT for short) (Baker, Xiang & Atkinson, 2017) are increasingly developed as systems of microservices: independently executable components that communicate *via* message passing (Dragoni et al., 2017). These systems typically have to deal with the continuous acquisition, processing, and distribution of semi-structured data. Over the last decade, the need for such data handling has contributed significantly to the adoption of document-oriented querying frameworks (Leavitt, 2010), like the MongoDB aggregation framework (MongoDB Inc., 2022)—and especially so in settings where Cloud Computing (Armbrust et al., 2010) is involved as well.

Recently, the necessity for careful data handling and the introduction of data protection regulations like the GDPR (Van Alsenoy, 2019) has highlighted the importance of handling ephemeral data (Shein, 2013). That is, in order to limit the circulation of data, applications should quickly process information without relying on persistency.

Ephemeral data handling is particularly relevant in scenarios where privacy is important (Mostert et al., 2016), for example eHealth (electronic systems that support healthcare) (Baker, Xiang & Atkinson, 2017), because it ensures by construction that data is automatically discarded unless the developers manually specifies otherwise. However, collecting and querying data with general-purpose languages in these contexts is often time consuming and error-prone (Reda, Piccinini & Carbonaro, 2018; Ma, Wang & Chu, 2013). In particular:

1. The implementation of query pipelines can quickly become complicated without proper abstractions.
2. Data might come from heterogeneous sources and in different data formats.

To solve the first issue (querying), developers typically include in their systems components that offer dedicated query languages (Cheney, Lindley & Wadler, 2013). For semi-structured data, a popular approach is to store data in a MongoDB instance (MongoDB Inc., 2018b), and then to use the MongoDB aggregation framework to perform queries.

As for the second issue (heterogeneity), developers can get support from programming languages or frameworks in which programs abstract from the concrete representation of data on the wire. Then, data is converted into the appropriate format and communicated through the appropriate protocol at runtime. Jolie is a (micro)service-oriented language designed to offer this capability (Montesi, Guidi & Zavattaro, 2014). A Jolie service can type, communicate, and manipulate semi-structured data under a unifying model that abstracts from data formats and communication protocols. Then, the program can be reused with different deployment instructions, which inform the Jolie engine of how data should be formatted (binary representations, JSON, XML, *etc*.) and communicated (using HTTP, SOAP, *etc*.) (Montesi, 2016). Jolie instructions can further be composed in workflows (Gabbrielli, Giallorenzo & Montesi, 2014); a feature that simplifies the programming of data collection and distribution in IoT and edge environments (Gabbrielli et al., 2019).

### The Problem

Ideally, a tool for ephemeral data handling in microservices would give us the best of the MongoDB aggregation framework and of the Jolie programming language: a query framework designed for semi-structured data and a language for working in heterogeneous environments.

An obvious attempt at achieving what we want is to just “stick together” MongoDB with Jolie, in the sense of deploying a Jolie service in the company of a MongoDB instance. Unfortunately, this approach runs into issues:

Dependency
: An external Database Management System (DBMS) like MongoDB is an additional standalone component that needs to be installed, deployed, and maintained. As with any software dependency, this exposes the applications to challenges of version incompatibility (Jang, 2006).
Security
: The companion DBMS is subject to weak security configurations (Brian Krebs, 2017) and query injections, increasing the attack surface of the application. This is a typical problem in microservices-with-database deployments where usually the microservice composes queries by assembling external inputs as strings, which is the main vector for query injections (Ron, Shulman-Peleg & Puzanov, 2016).
Inconsistency
: The key features of the MongoDB aggregation framework have only recently been formally understood, and some present idiosyncrasies related to implementation that do not make sense for a clean, abstract model (Botoeva et al., 2018).
Performance
: The communication channel between the MongoDB instance and the Jolie service can become a bottleneck, introducing the usual performance issues of database connections (Visveswaran, 2000). This is common in microservices-with-database scenarios where the overheads of establishing database connections can limit the performance of the whole component (and techniques, like managing pools of persistent database connections, are partial solutions (Visveswaran, 2000) that make the logic of the microservices more involved). Data format conversions in these communications contribute to overhead as well, together with the necessary measures to ensure ephemerality (post-query data deletion).

### Our solution

We propose the integration of relevant MongoDB data-query operators in Jolie. Our solution avoids the issues above: **Dependency**, since there is not anymore a database that we need to install and maintain; **Security**, because shedding the database removes risks from weak security configurations and, since the queries are part of the language (and not simply strings that we forward to the database engine), we also lower the exposition to query injections; **Inconsistency**, by building upon previous work on the formalisation of a consistent data-query theory of MongoDB (Botoeva et al., 2016); **Performance**, since there is no database involved, we avoid the overhead of: passing the data to and from the database; possible data-format conversions; bottlenecks due to pools of database connection channels (and possible bugs linked to their management), and of ensuring ephemerality.

### This article

We present two main contributions. The first one is a formal model of a query language for semi-structured data, called Tquery. The second is an implementation of Tquery, called Jolie/Tquery, which is the first query framework designed for ephemeral data handling in microservices. Jolie/Tquery addresses the problem by joining the benefits of Jolie and of the MongoDB aggregation framework: data can be collected from heterogeneous sources and then be queried in local memory by using pipelines of operations on semi-structured data.

The development of Jolie/Tquery is inspired by cleanroom software engineering. In particular, we have implemented our framework from scratch, starting from a formal model of its operators and their semantics. Our main contributions are described in the following.

**Formal Specification** We define Tquery, a theory for querying semi-structured data compatible with Jolie. Tquery provides the key operators of the MongoDB aggregation framework (match, unwind, project, group, and lookup), but reformulated for Jolie data structures and their accompanying syntax of paths for data traversal.

**Implementation** We develop Jolie/Tquery, an implementation of Tquery in the form of a Jolie package that can be used in services. Jolie/Tquery is lightweight: the entire compiled package consists of less than 100 kb. The implementation consists of two parts: an Application Programming Interface (API) to construct and run query pipelines, which defines the syntax of Tquery operators in terms of Jolie types; and an implementation of the API that follows the semantics given in Tquery. Jolie comes with an engine that supports implementing Jolie APIs with different languages (Montesi, 2016). In our case, Jolie/Tquery is implemented in Java. Jolie applications can use Jolie/Tquery by passing data in local memory and using native Jolie structures, which avoids the aforementioned issues. At the same time, Jolie applications can use Jolie’s capabilities for integrating with heterogeneous components to collect and distribute data.

**Evaluation** We illustrate the expressivity of Jolie/Tquery by using it to implement a use case from eHealth: a detection system for encephalopathy based on a proposal by Vigevano & Liso (2018). We then carry out microbenchmarks to validate the expectation that using Jolie/Tquery, being an in-memory query framework, outperforms using an external database management system (MongoDB specifically).

The article is structured as follows. ‘Related Work’ covers the related work. ‘Overview and Running Example’ illustrates the Tquery with the running example from the eHealth. ‘The Tquery Formalisation’ introduces formalisation of Tquery. ‘Implementation’ presents implementation of Tquery as a microservice written in Jolie programming language. ‘Benchmarks’ provides the benchmarks comparing the Tquery with the MongoDB. ‘Discussion and Conclusion’ drives conclusions.

This is the journal version of Giallorenzo et al. (2019), a short conference article where we presented preliminary ongoing work about the implementation of Jolie/Tquery.

## Related Work

Jolie/Tquery is the first implementation from scratch of a formally-specified, document-oriented query framework. Our formal model, Tquery, stands on the shoulders of MQuery (Botoeva et al., 2018), the first formal model of query operators for JSON data structures. MQuery formalises the key operators of the MongoDB aggregation framework, dispensing from some unnecessary idiosyncrasies that can lead to counterintuitive behaviour. Tquery inherits this good feature—the reader interested in the technical differences w.r.t. the MongoDB aggregation framework can consult (Botoeva et al., 2016, Appendix C). The key difference between this work and Botoeva et al. (2018) is that Tquery comes with an implementation, whereas Botoeva et al. (2018) investigated the theoretical expressivity of the MQuery operators w.r.t. relational algebra and their complexity. Tquery adopts the same operators but reformulated to be compatible with the Jolie data model (by adopting arrays instead of unordered forests for document collections). The semantics of our operators is also specified differently: while Tquery’s operators follow the same intuition of the operators in MQuery, we give our semantics specifying how operations can be computed. For example, we do not rely on existential quantification and all our definitions are given by recursion on the structures of inputs. We believe that formalisation efforts like MQuery and Tquery are important: during the development of our implementation, we found having a reference formal model helpful to clarify the expected behaviour of operators and what tests we should write.

Jolie has been used in several domains that require ephemeral data handling, including smart mobility (Callegati et al., 2017), IoT (Gabbrielli et al., 2018), integration components in document management systems (Maschio, 2019), and media content (Maschio, 2017). However, due to the lack of an appropriate query framework, the query logic has been implemented manually with a general-purpose computation language (the computation layer of Jolie). Because it guarantees that data gets discarded (ephemerality) and it provides an expressive set of compositional query operators, Jolie/Tquery offers a better alternative for writing data-intensive Jolie microservices. Moreover, since every Jolie program is a composition of services, adapting a program to offload parts of its computations to remote nodes is simple (it mainly regards the reconfiguration of how services are deployed). This, in unison with the fact that Jolie/Tquery operators are stateless, simplifies the task of splitting Jolie/Tquery heavy-weight or computation-intensive queries over multiple nodes.

Other solutions that offer semi-structured data querying in separate services include MongoDB (MongoDB Inc., 2018b) and CouchDB (Apache, 2005); however, these are DB-based solutions that fall into the category of deployments that we deem unfit for the case of ephemeral data-handling. Moreover, these do not come with a formal model which, *e.g.*, one can use to reason about the semantics of the implementation and to check its consistency (like Botoeva et al. (2016) demonstrated for MongoDB).

There exist works on the integration of relational query frameworks with general-purpose programming languages, including: object-relation mapping frameworks (ORMs), which map objects to database entities (Fussel, 1997); Opaleye, a Haskell DSL for generating PostgreSQL commands (Ellis, 2014); and LINQ (Meijer, Beckman & Bierman, 2006), which provides query operators targeting SQL tables and XML structures for .NET languages. Tquery could be a reference to implement similar frameworks for semi-structured data in these languages. A convenient feature of Jolie/Tquery is that all its queries can work with any data format that Jolie can handle: Jolie automatically converts data in different formats (including JSON, XML, and some binary formats) to its abstract data model (Montesi, Guidi & Zavattaro, 2014; Montesi, 2016).

As we are going to exemplify in the next section, a typical use case for semi-structured data handling and Jolie/Tquery is the reactive processing of events. Stream-processing languages have been explored for similar tasks, but they feature different kinds of primitives and are usually not based on semi-structured data.

The landscape of stream-processing languages is quite wide, *e.g.*, data-centric (Chen et al., 2000; Barbieri et al., 2009), time- or hardware-constrained execution-centric (Caspi et al., 1987; Hirzel, Schneider & Gedik, 2017; Tommasini et al., 2019), focussed on the relational- or document-oriented (Chen et al., 2000; Diao et al., 2002; Mendell et al., 2012) approach. In particular, SQL-based stream-processing languages (Esteves et al., 2017; Babu & Widom, 2001) recently gained popularity in industry (thanks to the familiarity of programmers with the SQL language), with commercial tools such as Apache Flink (Apache, 2022a), Apache Kafka (KSQL) (Narkhede, 2017), Apache Samza (Apache, 2022b), Apache Storm (Apache, 2022c), WSO2 Stream Processor (WSO2, 2022), Siddhi (Siddhi Streaming SQL) (Siddhi, 2022). We deem StreamQL (Kong & Mamouras, 2020) the work closest to Jolie/Tquery. This is a query language for efficiently processing IoT data streams. The StreamQL Engine is implemented as a lightweight Java library and does not depend on the external engine. However, StreamQL is a functional language that is based on formal semantics residing on the class of monotone functions over streams. It works with the typical functional primitives on list-based data, supporting a variety of operators that simplify stream-processing at the level of data aggregation (filtering, windowing, etc.) and data-flow control (*e.g.*, parallel composition). StreamQL does not handle explicitly semi-structured document-oriented data and requires additional processing for data translation, while Jolie/Tquery handles it natively(tree-shaped data simplifies integration with Jolie). Unlike Jolie/Tquery, StreamQL has built-in primitives for temporal control typical for data streaming languages. In Jolie/Tquery time contracts can be implemented by adding information to the data structures and need to be managed explicitly by the programmer. Widening our scope, we deem two works, CQL (Arasu, Babu & Widom, 2006) and EQL (Elasticsearch, 2022), close to Jolie/Tquery. CQL is a declarative streaming SQL-based query language, implemented in the STREAM DSMS (Arasu et al., 2016) with data captured with sliding windows (Babcock et al., 2002) based on time-(*e.g.*, update the data every 30 s) and data-related conditions (*e.g.*, capture the data as soon as it arrives). EQL (Elasticsearch, 2022) is an event-based data manipulation library developed in Python. Similarly to CQL, EQL expects data to follow an event-oriented schema. Interestingly, EQL provides a query-composition operator similar to the one provided by Jolie/Tquery (see ‘Extending Jolie/Tquery with query pipelines’). Both CQL and EQL, being SQL-based, work on tuples of data rather than semi-structured documents as Jolie/Tquery does—e.g., one needs to convert a JSON document into tuples of data before using CQL/EQL.

Finally, Ballerina (Oram, 2019) is a language for the development of microservices close to Jolie, developed by WSO2, that equips SQL-like query operators to process data and events. The differences with Jolie/Tquery include the relational nature of the operators, which requires the user to translate values between document- and tuple-shaped data when applying/using the data from the queries, and the lack of a formal reference.

## Overview and Running Example

In this section, we illustrate our proposal with an eHealth use case, showing the definition of a diagnostic algorithm as a composition of Tquery’s operators. We deem this area of application apt to illustrate Tquery for two main reasons.

First, since medical diagnostic algorithms are usually expressed through declarative or high-level imperative instructions, having high-level, declarative operators for data handling narrows the gap between definitions and implementations and helps in both translating and checking their correctness. Indeed, more and more studies emerged proposing non-intrusive, affordable yet accurate diagnostic systems based on data collected from heterogeneous sources such as user-inputted data, smartphones, wearables, and cameras (Purohit et al., 2020). An emblematic example of this phenomenon is the recent proposal by Hirten et al. (2020), where the authors defined and demonstrated the efficacy of a diagnostic algorithm to identify and predict SARS-CoV-2 (aka COVID-19) infections, reporting promising predictive ability to identify infection days before the diagnosis through nasal-swab testing. Here, we focus on a simpler-yet-comprehensive diagnostic algorithm defined by Vigevano & Liso (2018) to detect cases of encephalopathy.

Second, the inherent ephemerality of Tquery programs caters to the principles of secrecy and obliviousness of data—the data handled by a Tquery program is automatically deleted from memory—in the healthcare sector. This approach is frequently summarised by the motto “the data never leave the hospital” and it is compliant with the current regulations on data protection (*e.g.*, GDPR (Rose, 2014)).

In the remainder of the article, we use the diagnostic algorithm by Vigevano & Liso (2018) to illustrate the formal semantics of Tquery. Here, we focus on the overall definition of the parts of the algorithm and how we can map them to a combination of Tquery operators acting on and merging data from different sources. Then, in ‘The Tquery Formalisation’, we return on the single instructions that make up the algorithm presented here and show the step-by-step output of Tquery operators, following from the specification of their semantics.

### An encephalopathy diagnostic algorithm

Taking inspiration from Vigevano & Liso (2018), we focus on the aggregation of two early markers to detect encephalopathy: fever in the last 72 h and lethargy in the last 48 h. Those data are collectable by commercially-available smart-watches and smart-phones (Bunn et al., 2018): body temperature and sleep quality.

Tquery defines operators over tree-like data structures, formally defined in ‘Data structures: trees and paths’. To keep this example compact, it is sufficient that the reader has some familiarity with data formats like XML (Bray et al., 2000) and JSON (Crockford, 2006) documents. Specifically, here we use a subset of the JSON format where a tree is represented by a pair of brackets {} which enclose a set of ordered pairs, each linking a label (unquoted) to an array, whose content is enclosed within square brackets [ ] . Arrays can either contain trees or primitive values (string, integer, *etc*.).

As an example of the format above, we report in Listing 1 code snippets exemplifying the shape of the two data structures used in the example: the first (Lines 2–5) carries the temperature and heart-rates, the second holds the sleep logs (Lines 7–16) (Thurman et al., 2018).

At Lines 2–5, for each — date— we have an array of detected temperatures (— t—) and heart-rates (— hr—). At Lines 7–16, to each year (— y—) it corresponds an array of monthly (— M—) measures, to a month (— m—), an array of daily (— D—) logs, and to a day (— d—), an array of logs (— L—), each representing a sleep session with its start (— s—), end(— e—), and quality (— q—).

 
 
 
 _______________________________________________________________________________________________________________________ 
 
 1 //  representation  of  the  tmp  data  structure 
  2 [ { date : [ 20201127 ] , t : [ 37 ] , hr : [ 64 ] } , 
  3  { date : [ 20201128 ] , t : [ 36 ] , hr : [ 66 ] } , 
  4  { date : [ 20201129 ] , t : [ 36 ] , hr : [ 65 ] } , 
  5  { date : [ 20201130 ] , t : [ 37 ] , hr : [ 67 ] } ] 
  6 //  representation  of  the  sl  data  structure 
  7 [ { y : [ 2020 ] , 
  8  M : [ { m : [ 11 ] , 
  9  D : [ { d : [ 27 ] , L : [ { s : [ ' 23:33 ' ] , e : [ ' 07:04 ' ] , q : [ ' poor ' ] 
  } ] } , 
  10  { d : [ 28 ] , L : [ { s : [ ' 21:13 ' ] , e : [ ' 09:34 ' ] , q : [ ' good ' 
  ] } ] } , 
  11  { d : [ 29 ] , L : [ { s : [ ' 21:01 ' ] , e : [ ' 03:12 ' ] , q : [ ' good ' 
  ] } , 
  12  { s : [ ' 03:36 ' ] , e : [ ' 09:58 ' ] , q : [ ' good ' 
  ] } ] } , 
  13  { d : [ 30 ] , L : [ { s : [ ' 20:33 ' ] , e : [ ' 01:14 ' ] , q : [ ' poor ' 
  ] } , 
  14  { s : [ ' 01:32 ' ] , e : [ ' 06:15 ' ] , q : [ ' good ' 
  ] } ] } 
  15  ] } ] } ] 
  16 } ] 
 Listing 1: Snippets of biometric (Lines 2–5) and sleep logs (Lines 7–16) data.    

To implement the algorithm for detecting encephalopathy, we need to integrate with two functionalities provided by the Hospital IT infrastructure: — detectFever— and — detectEncephalopathy—. The former accepts data of the shape:

 
 
 
_______________________________________________________________________________________________________________________ 
 
   [ { t : [ 36 , 37 , 38 ] , patient_id : [ ' xyz '  ] } ] 
where t contains the array of measured temperatures in the last three 
days and patient_id the identifier for the patient in the Hospital 
IT infrastructure.  The latter accepts the format: 
 
 [ { temperatures : [ 36 , 37 , 38 ] , patient_id : [ ' xyz '  ] , 
 quality : [ ' good ' , ' poor '  ] } ]    

where — temperatures— contains the measured temperatures in the last three days, — patient˙id— contains the identifier for the patient in the Hospital IT infrastructure (essentially, this is the same data found, respectively, under — t— and — patient˙id— issued to the — detectFever— functionality), and — quality— contains the recorded quality of sleep in the last two days.

Our focus in the last part of this section is to describe—by means of the Tquery operators—a program that manipulates the biometric and sleep logs data in Listing 1 to integrate the functionalities — detectFever— and — detectEncephalopathy— and implement the diagnostic algorithm.

### An overview of the Tquery operators

Before presenting the diagnostic algorithm, we give a brief and informal description of the shape and effect of each Tquery operator (presented formally in ‘The Tquery Formalisation’), as a reference to integrate the description of the example.

• the *match* operator *μ*, given an array and a match criterion returns the elements of the array that satisfy the criterion, in their relative order from the input;
• the *unwind* operator *ω* takes as inputs an array and a path *p*.^1^ 1Intuitively, a path is a sequence of node labels of the shape $\underline{A.B.C}$. Formally, cf. ‘Data structures: trees and paths’.The result of the application is a new array containing the “unfolding” of the input array under the path, *i.e.,* where we take each element *e* from the input array, we find all values under *p* in *e* and, for each value, we include in the new array a copy of *e* except it holds only that single value under *p*;
• the *project* operator *π*, given an array and a projection expression, it returns a copy of the original array with each element updated by the projection expression. Projection expressions can move/rename and remove sub-parts from the elements, as well as insert new ones;
• the *group* operator *γ* takes as inputs an array and two lists of paths: a grouping list and an aggregation list. The result of the application is a new array where each element has two properties: *(i)* it includes the combinations of distinct values from the set of values found under the grouping paths among the elements in the input array; *(ii)* it aggregates all the values found under the aggregation paths among the elements in the input array which have been grouped by the same combination of values;
• the *lookup* operator *λ* joins two arrays, a “source” and an “adjunct” one, according to the correspondence of values in their elements with respect to a source path and an adjunct path. Besides those inputs, the operator requires a “destination” path. The application of the operator returns a new array that contains all the elements resulting from merging each element *e*_*s*_ in the source array with the elements *e*_*a*_ in the adjunct array such that *e*_*s*_ and *e*_*a*_ hold the same values under the respective source and adjunct paths. The resulting array contains all the elements from the source, each updated to include, under the provided destination path, all path-matching elements from the adjunct array.

### Implementing the diagnostic algorithm with Jolie and Tquery

Here, on the data structures and operators described above, we define a Jolie microservice (reported in Listing 2), which implements the handling of the data and the workflow of the use-case diagnostic algorithm.

The example is broad enough to let us illustrate all the operators in Tquery and to represent a real-world workflow, where, besides implementing the algorithm of interest, we manipulate the data for system integration (*e.g.*, by reshaping the data structures to fit the service APIs we need to invoke). Note that, while in Listing 2 we hard-code some data (*e.g.*, integers representing dates like — 20201128—) for presentation purposes, we would normally use parametrised variables.

Since we follow a formalisation-first approach to present Tquery, in Listing 2 we interleave runnable Jolie code with the formal definition of the application of the involved Tquery operators. When doing so, we use the highlighted, algorithmic notation ⋯←⋯. After having defined the formal semantics of the operators in ‘The Tquery Formalisation’, we will present the actual implementation of the example in ‘Implementation’ using our implementation of the Tquery operators in Jolie.

Note also that, while variables of the form — patientData— and — tmp— in Listing 2 conveniently resemble variable symbols as found in Java or C, they are actually path applications on the state of a Jolie program, which is a tree. Hence, the meaning of — tmp— reads “get the structure pointed by path $\underline{tmp}$ in the current state of the program”. In the example, when assigning and passing values, we use the notation — a— and — b.c— to indicate the path traversal and retrieval of the structure pointed by the respective paths $\underline{a}$ and $\underline{b.c}$ on the state of the Jolie program. We instead use the notation $\underline{a}$ and $\underline{b.c}$ to indicate the passing of paths as parameters of Tquery operators.

We now describe the diagnostic algorithm and how we use the Tquery operators to implement it.

In Listing 2, at Line 1 we find the Jolie code of a request to an external service, provided by the HospitalIT infrastructure. The service offers the functionality — getPatientPseudoID— which, given some identifying — patientData— (acquired earlier), provides a pseudo-anonymised identifier—needed to treat sensitive health data—saved in variable — pseudoID—.

At Line 2 we retrieve in the variable — credentials— the keys to access the physiological sensors of the patient to obtain the biometric data (Listing 1, Lines 1–5) from the SmartWatch of the patient, by invoking the functionality — getMotionAndTemperature— and storing the result in — tmp—.

At Lines 3–5 we use the Tquery operators *μ*, *γ*, and *π* to extract the recorded temperatures of the patient in the last 3 days/72 h. At Line 3 we use the match operator *μ* to filter all the entries of the biometric data, keeping only those of the last 72 hours/3 days. At Line 3, we aggregate the result of the *μ* (which replaced the previous value under — tmp—) under the path $\underline{t}$ and discard the others. At Line 5, we use the project operator to include in — tmp— the identifier of the patient, under $\underline{patient\text{_}id}$.

At Line 6, we call the external functionality — detectFever— to analyse the temperatures and check if the patient manifested any fever, storing the result in — hasFever—.

 
 
 
  _____________________________________________________________________________________________________________________ 
 
  1 getPatientPseudoID @ HospitalIT ( patientData  ) ( pseudoID  ) 
   2 getMotionAndTemperature @ SmartWatch ( credentials  ) ( tmp  ) 
   3 tmp ← μ ( tmp , ( date  =20201128 ∨ date  =20201129 ∨ date  =20201130) 
   ) 
   4 tmp ← γ ( tmp , ( t  ) , () ) 
   5 tmp ← π ( tmp , ( t  , pseudoID  〈〉 patient id  ) ) 
   6 detectFever @ HospitalIT ( tmp  ) ( hasFever  ) 
   7 if ( hasFever  ) { 
  8  getSleepPatterns @ SmartPhone ( credentials  ) ( sl  ) 
   9  sl ← ω ( sl , M.D.L  ) 
   10  sl ← π ( sl , ( y  〈〉 year  , M.m  〈〉 month  , M.D.d  〈〉 day  , 
   M.D.L.q  〈〉 quality  ) ) 
   11  sl ← μ ( sl , ( year  =2020 ∧ month  =11 ∧ ( day  =29 ∨ day  =30) ) ) 
   12  sl ← γ ( sl , ( quality  ) , () ) 
   13  sl ← π ( sl , ( quality  , pseudoID  〈〉 patient id  ) ) 
   14  bs ← λ ( sl , patient id  , tmp , patient id  , temperatures  ) 
   15  bs ← π ( bs , ( quality  , patient id  , temperatures.t  〈〉 temperatures  ) ) 
   16  detectEncephalopathy @ HospitalIT ( bs  ) 
   17 } 
      Listing 2: Snippet implementing the diagnostic algorithm.    

After the analysis on the temperatures, — if— the patient — hasFever— (Line 7), we continue testing for lethargy. To do that, at Line 8 we follow the same strategy described for Line 2 to pass the — credentials— to the functionality — getSleepPatterns—, used to collect the sleep logs of the patient from her — SmartPhone— in — sl—.

Then, since the sleep logs are nested under years, months, and days, to filter the logs relative to the last 48 hours/2 days, we first flatten the structure through the unwind *ω* operator applied on the path $\underline{M.D.L}$ (Line 9). For each nested node (separated by the dot in the path), *ω* generates a new data structure for each element in the array reached by that node. Concretely, the array returned by the *ω* operator at Line 9 contains each sleep log associated with the full date of the recording (year, month, and day), as shown below.

 
 
 
   _______________________________________________________________________________________________________________________ 
 
   [ { y : [ 2020 ] , M : [ { m : [ 11 ] , D : [ { d : [ 27 ] , L : [ { s : [ ' 23:33 ' ] , ... } ] } ] } ] 
  } , 
  { y : [ 2020 ] , M : [ { m : [ 11 ] , D : [ { d : [ 28 ] , L : [ { s : [ ' 21:13 ' ] , ... } ] } ] } ] 
  } , 
  { y : [ 2020 ] , M : [ { m : [ 11 ] , D : [ { d : [ 29 ] , L : [ { s : [ ' 21:01 ' ] , ... } ] } ] } ] 
  } , 
  { y : [ 2020 ] , M : [ { m : [ 11 ] , D : [ { d : [ 29 ] , L : [ { s : [ ' 03:36 ' ] , ... } ] } ] } ] 
  } , 
  ... ]    

Given the new shape of — sl—, at Line 10 we modify the data structure with the project operator *π* to simplify the subsequent commands: we rename the node $\underline{y}$ to $\underline{year}$, we move and rename the node $\underline{M.m}$ to $\underline{month}$ (bringing it at the same nesting level of $\underline{year}$); similarly, we move $\underline{M.D.d}$, renaming it $\underline{day}$, and we move $\underline{M.D.L.q}$ (the log of the quality of the sleep), renaming it $\underline{quality}$—$\underline{M.D.L.s}$ and $\underline{M.D.L.e}$, not included in the parameters of the projection, are discarded.

On the obtained structure, we filter the sleep logs relative to the last 48 h with the match operator at Line 11.

At Line 12 we use the grouping operator *γ* to aggregate the $\underline{quality}$ of the sleep sessions recorded in the same day and discarding the nodes $\underline{day}$, $\underline{month}$, and $\underline{year}$.

At Line 13 we project within the — sl— data structure the — pseudoID— of the patient under node *patient*_*id*. That value is used at Line 14 to join, with the lookup operator *λ*, the obtained sleep logs with the previous values of temperatures (— tmp—). Lastly, we prepare the data structure to be submitted for analysis. We do this at Line 15 by keeping the paths $\underline{quality}$ and $\underline{patient\text{_}id}$ in — bs— and by moving the nested temperatures ($\underline{temperatures.t}$) under the path $\underline{temperatures}$—this is required by the interface of — detectEncephalopathy—, which we invoke passing the resulting (— bs—) data structure.

## The Tquery Formalisation

In this section, we report the formalisation of Tquery. Besides providing a general, mathematical reference, the formalisation guides the implementation of our Jolie framework, presented in ‘Implementation’. Tquery is inspired by MQuery (Botoeva et al., 2018), a sound variant of the MongoDB Aggregation Framework (MongoDB Inc., 2018a); the most popular query language for NoSQL data handling.

In our formal development, we favour a theory-to-practice strategy to avoid inconsistent or counter-intuitive query behaviours, which is one of the significant drawbacks of the MongoDB Aggregation Framework implementation (Botoeva et al., 2018). Moreover, we consider the formalisation as a blueprint for implementors and thus we strive for a balance between abstraction and technical involvement: *(i)* we adopt constructive semantics definitions rather than declarative ones, since the former are more amenable to imperative implementations, and *(ii)* we define our semantics on trees rather than on sets (as done in Botoeva et al. (2018)), since the former is the data structure handled by the developers and their users.

### Data structures: trees and paths

We start by defining trees and the primitives on which we define the semantics of Tquery.

We denote trees with *t*. A tree contains two elements: (i) a *root* value that we denote with *b*, which holds basic values (Booleans, integers, and strings) or the null value *υ*; (ii) a set of pairs $~{ k:a~} $, where each pair associates a *key k* to an *array* of trees $a$. Formally:

\begin{eqnarray*}t\,::=\,b~\text{\{}{k}_{i}:{a}_{i}~\text{\}}\qquad \qquad a\,::=\,~\text{[}{t}_{1},\ldots ,{t}_{n}~\text{]} \end{eqnarray*}

We indicate with *k*(*t*) the extraction of the array pointed by the label *k* in *t*: if *k* is present in *t* we retrieve the related array, otherwise we return the null array *α* (different from the empty array, instead denoted with [ ] ). Formally:

\begin{eqnarray*}k(b~\text{\{}{k}_{i}:{a}_{i}~{\text{\}}}_{i})= \left\{ \begin{array}{@{}ll@{}} \displaystyle a &\displaystyle \text{if}(k:a)\in ~\text{\{}{k}_{i}:{a}_{i}~{\text{\}}}_{i}\\ \displaystyle \alpha  &\displaystyle \text{otherwise} \end{array} \right. \end{eqnarray*}

We assume the range of a given array *a* to run from the minimum index (one) to the maximum, that corresponds to its cardinality, denoted with #*a*. We indicate the extraction of the tree *t* at index *i* in array *a* with the index notation *a*[*i*], *i.e.,* *a*[*i*] = *t*. In case *a* contains an element at index *i* we retrieve it, otherwise, we retrieve the null tree, denoted with *τ*. Formally:

\begin{eqnarray*}a[i]= \left\{ \begin{array}{@{}ll@{}} \displaystyle {t}_{i} &\displaystyle \text{if}a=~\text{[}{t}_{1},\ldots ,{t}_{n}~\text{]}\wedge 1\leq i\leq n\\ \displaystyle \tau  &\displaystyle \text{otherwise} \end{array} \right. \end{eqnarray*}

We define the array concatenation operator, denoted with ::, such that  [*t*_1_, …, *t*_*n*_ ] =  [*t*_1_ ]::…:: [*t*_*n*_ ]. Given two arrays *a*′ and *a*^′′^, the concatenation *a*′::*a*^′′^ returns an array *a* of size #*a* = #*a*′ + #*a*^′′^ where elements *a*[1], …, *a*[#*a*′] correspond point-wise to elements *a*′[1], …, *a*′[#*a*′] and elements *a*[#*a*′ + 1], …, *a*[#*a*′ + #*a*^′′^] correspond point-wise to elements *a*^′′^[1], …, *a*^′′^[#*a*^′′^].

We define paths to express tree traversal, ranged over by *p*. Paths are concatenations of expressions, indicated with *e* (which we omit to define since orthogonal to Tquery), closed by the sequence termination *ɛ*. Formally:

\begin{eqnarray*}p\,::=\,\underline{e.}p\;\mid \;. \end{eqnarray*}

When possible, we omit to indicate sequence terminations *ɛ* in paths and we slightly abuse the notation by indicating the components of paths like $\underline{e.}p$ as *e*.*p* to keep a lightweight notation—this does not make the notation ambiguous since path concatenation is always contextually distinct.

The application of a path *p* to a tree *t*, written [[*p*]]^*t*^, returns an array that contains the sub-trees reached traversing *t* following *p*. To define [[*p*]]^*t*^, we introduce the notation *e*↓*k*, read “*e* evaluates to *k*”, and use it to indicate that the evaluation of the expression *e* in a path *p* results in the label *k*. Path application [[*p*]]^*t*^ neglects array indexes, *i.e.,* for *p* = *e*.*p*′, such that *e*↓*k*, we apply the sub-path *p*′ to all trees in the array pointed by *k* in *t* and concatenate all their results keeping their relative order—the resulting array can concatenate null arrays *α* too, as a result of applying the path on some (sub)trees that do not contain all nodes present in *p*.

\begin{eqnarray*}[ [\,p\,] ]^{t}= \left\{ \begin{array}{@{}ll@{}} \displaystyle [ [\,{p}^{{^{\prime}}}\,] ]^{{t}_{1}}::\ldots ::[ [\,{p}^{{^{\prime}}}\,] ]^{{t}_{n}} &\displaystyle \text{if}p=e.{p}^{{^{\prime}}}\wedge e \downarrow  k\wedge k(t)=~\text{[}{t}_{1},\ldots ,{t}_{n}~\text{]}\\ \displaystyle ~\text{[}t~\text{]} &\displaystyle \text{if}p=\\ \displaystyle \alpha  &\displaystyle \text{otherwise} \end{array} \right. \end{eqnarray*}

We illustrate the path application with the example below, where *t*_1_ = sl[1], *i.e.,* it is the first (and only) element in the — sl— data structure represented at Lines 7–16 of Listing 1. From now on, in the examples, we adopt the formal representation of trees defined at the beginning of the section.

 
 
 
__________________________________________________________________________________________________ 
 
  [[M.m.D.d]]t1 [27{}, 28{}, 29{}, 30{}]    

In the remainder, to contract empty and null arrays, we assume the following structural equivalences when we perform array concatenations.

\begin{eqnarray*}\alpha \equiv \alpha ::\alpha \alpha ::[]\equiv []::\alpha \equiv []::[]\equiv []\alpha ::a\equiv a::\alpha \equiv a::[]\equiv []::a\equiv a \end{eqnarray*}

### Tquery operators

**Figure 1:**
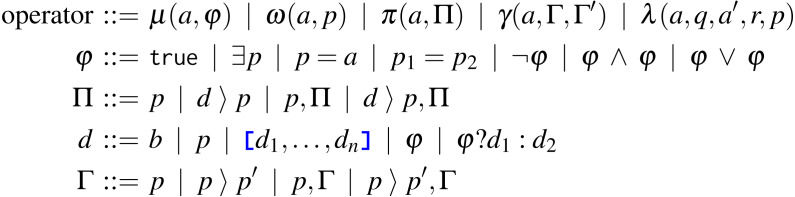
Syntax of Tquery.

In this section, we present each Tquery operator: examples of its usage, its formal syntax, and its semantics, with examples illustrating relevant steps. For reference, we report in Fig. 1 the syntax of the Tquery operators: *match* (*μ*), *unwind* (*ω*), *project* (*π*), *group* (*γ*), and *lookup* (*λ*). In the syntax, *a* denotes arrays, *b* denotes primitive values, and *p*, *q*, and *r* are paths. We define the parameters of the operators with four syntactic rules: *φ* for the match, Π and *d* for the project, and Γ for the group, explained in their relative sections.

#### The match operator


\begin{eqnarray*}\mu (a,\varphi )\qquad \varphi \,::=\,true\;\mid \;\exists p\;\mid \;p=a\;\mid \;{p}_{1}={p}_{2}\;\mid \;\neg \varphi \;\mid \;\varphi \;\wedge \;\varphi \;\mid \;\varphi \;\vee \;\varphi \end{eqnarray*}

The purpose of the *match* operator is to select trees in an array *a* according to a criterion *φ*, which can be (from left to right): (i) the Boolean truth, (ii) the existence of a path *p* in *t*, (iii) the equality between the application of a path *p* on *t* and a given array *a*, (iv) the equality between the applications of two paths *p*_1_ and *p*_2_ on *t*, and the logic connectives (v) negation, (vi) conjunction, and (vii) disjunction.

**Example** Here and in the following sections, we draw our examples from Listing 2. There, we see the match operator used twice: the first at Line 3 and the second at Line 11. Here, we focus on the example at Line 3. We comment the execution of Line 11 in ‘The group operator’, since we use it to filter out the unnecessary values from the — sl— data structure before the application of the *group*.

At Line 3, we use a match to filter — tmp— from those trees that do not correspond to the time range of interest. For convenience, we report Line 3 of Listing 2 in the snippet below.

 
 
 
  ____________________________________________________________________________________________________________ 
 
  3 tmp ← μ ( tmp , ( date  =20201128 ∨ date  =20201129 ∨ date  =20201130)  )    

The execution takes as input the data structure — tmp— presented in Listing 1 and assigns to it the resulting data structure:


 
 
 
  ______________________________________________________________________________________________________________________________________ 
 
  [ υ { date : [ 20201128 { } ] , t : [ 36 { } ] , hr : [ 66 { } ] } , 
  υ { date : [ 20201129 { } ] , t : [ 36 { } ] , hr : [ 65 { } ] } , 
   υ { date : [ 20201130 { } ] , t : [ 37 { } ] , hr : [ 67 { } ] } ]    


**Semantics** When applied to an array *a*, the match operator returns a new array in the shape of *a* but including only its elements that satisfy *φ*. If no element matches the criterion (and also in the case that *a* = *α*), the operator returns an empty array [ ] .

\begin{eqnarray*}\mu (\alpha ,\varphi )=[] \mu ([t]::a,\varphi )= \left\{ \begin{array}{@{}l@{}} \displaystyle [t]::\mu (a,\varphi )  & \text{if}t\models \varphi \\ \displaystyle \mu (a,\varphi )  & \text{if}\mathtt{#}a\gt 0\\ \displaystyle []  & \text{otherwise} \end{array} \right. \end{eqnarray*}

The semantics of *t*⊧*φ* is defined by the Boolean expressions below.

\begin{eqnarray*}t\models \varphi \text{holds iff} \left\{ \begin{array}{@{}l@{}} \displaystyle \varphi =true \\ \displaystyle \varphi =(\exists p)~\wedge ~[ [\,p\,] ]^{t}\not = \alpha  \\ \displaystyle \varphi =(p=a)~\wedge ~[ [\,p\,] ]^{t}=a \\ \displaystyle \varphi =({p}_{1}={p}_{2})~\wedge ~[ [\,{p}_{1}\,] ]^{t}=[ [\,{p}_{2}\,] ]^{t} \\ \displaystyle \varphi =(\neg {\varphi }^{{^{\prime}}})~\wedge ~t\not \models {\varphi }^{{^{\prime}}} \\ \displaystyle \varphi =({\varphi }_{1}\wedge {\varphi }_{2})~\wedge ~(t\models {\varphi }_{1}~\wedge ~t\models {\varphi }_{2}) \\ \displaystyle \varphi =({\varphi }_{1}\vee {\varphi }_{2})~\wedge ~(t\models {\varphi }_{1}~\vee ~t\models {\varphi }_{2})  \end{array} \right. \end{eqnarray*}

**Example: semantics** At Line 3 of Listing 2, the match evaluates all trees inside — tmp— and verifies which one of the sub-conditions hold for each element of — tmp—. In the case of — tmp—[1], the criterion is not satisfied and thus the value is discarded. Next, — tmp—[2] satisfies the first criterion  
 date = 20201128)    , — tmp—[3] satisfies the second criterion  
 
 
 
   \(\  date } =\  20201129 } \) , and — tmp—[4] satisfies the third criterion  
 
 
 
   \( date } =\  20201130 } \) .

#### The unwind operator


\begin{eqnarray*}\omega (a,p) \end{eqnarray*}

The purpose of the *unwind* operator is to unfold the elements of an array *a* under a given path *p*.

**Example** We exemplify the usage of *unwind* reporting Line 9 of Listing 2 in the snippet below and later showing the result of its application.

 
 
 
  _______________________________________________________________________ 
 
  9 sl ← ω ( sl , M.D.L  )    

The unwind operator takes as input the sleep logs — sl— (as retrieved from the invocation of the — getSleepPatterns— operation at Line 8, and represented at Lines 7–16 of Listing 1). In the snippet, we update the content of — sl— to contain the new data structure, shown below.

 
 
 
  ________________________________________________________________________________________________________________________ 
 
  [ υ { y : [ 2020 { } ] , M : [ υ { m : [ 11 { } ] , D : [ υ { d : [ 27 { } ] , 
   L : [ υ { s : [ ' 23:33 ' { } ] , e : [ ' 07:04 ' { } ] , q : [ ' poor ' { } ] 
   } ] } ] } ] } , 
   υ { y : [ 2020 { } ] , M : [ υ { m : [ 11 { } ] , D : [ υ { d : [ 28 { } ] , 
   L : [ υ { s : [ ' 21:13 ' { } ] , e : [ ' 09:34 ' { } ] , q : [ ' good ' { } ] 
   } ] } ] } ] } , 
   υ { y : [ 2020 { } ] , M : [ υ { m : [ 11 { } ] , D : [ υ { d : [ 29 { } ] , 
   L : [ υ { s : [ ' 21:01 ' { } ] , e : [ ' 03:12 ' { } ] , q : [ ' good ' { } ] 
   } ] } ] } ] } , 
   ... ]    

**Semantics** To define the semantics of the unwind operator *ω*, we introduce an auxiliary operator, called *unwind expansion operator* and we indicate it with ueo(*t*, *a*, *k*) (read “unwind *t* on *a* under *k*”). Informally, ueo(*t*, *a*, *k*) returns an array of trees with cardinality #*a* where each element has the shape of *t* except that label *k* points to the corresponding index-wise element in *a*.

Formally, given a tree *t*, an array *a*, and a key *k*:

\begin{eqnarray*}\mathsf{ueo}(t,a,k)= \left\{ \begin{array}{@{}ll@{}} \displaystyle ~\text{[}b \left( \left( ~\text{\{}{k}_{i}:{a}_{i}~{\text{\}}}_{i}\setminus ~\text{\{}k:k(t)~\text{\}} \right) \cup ~\text{\{}k:~\text{[}{t}^{{^{\prime}}}~\text{]}~\text{\}} \right) ~\text{]}::\mathsf{ueo}(t,{a}^{{^{\prime}}},k)&\displaystyle \text{if}a=~\text{[}{t}^{{^{\prime}}}~\text{]}::{a}^{{^{\prime}}}\\ \displaystyle &\displaystyle \wedge t=b~\text{\{}{k}_{i}:{a}_{i}~{\text{\}}}_{i}\\ \displaystyle ~\text{[}\text{]} &\displaystyle \text{otherwise}  \end{array} \right. \end{eqnarray*}

Then, the formal definition of *ω*(*a*, *p*) is

\begin{eqnarray*}\omega (a,p)= \left\{ \begin{array}{@{}ll@{}} \displaystyle \mathsf{ueo}(t,\omega ([ [\,\underline{k.}\,] ]^{t},{p}^{{^{\prime}}}),k)::\omega ({a}^{{^{\prime}}},p) &\displaystyle \text{if}p=e.{p}^{{^{\prime}}}\wedge e \downarrow  k\wedge a=~\text{[}t~\text{]}::{a}^{{^{\prime}}}\\ \displaystyle a &\displaystyle \text{if}p=\\ \displaystyle ~\text{[}\text{]} &\displaystyle \text{otherwise}  \end{array} \right. \end{eqnarray*}

Essentially, the semantics of the unwind operator follows two inductive directions: one on arrays and the other on paths. Hence, to simplify the explanation of the semantics, we describe it following a spatial interpretation of the two directions: the induction on arrays is the “breadth” of the expansion while the induction on paths represents its “depth”.

The first part of the breadth expansion corresponds to the induction over the array *a*, which results in the concatenation of the inductive application of the depth expansion of *p* over each element *t* of *a*. In turn, the depth expansion consists of a nested depth expansion with a breadth one. The depth expansion is represented by $\omega ([ [\underline{k.}] ]^{t},{p}^{{^{\prime}}})$, which corresponds to the application of the unwind operator with path *p*′—the suffix of *k* in *p*—and on the array of subtrees found in *t* under the current path fragment *k*. The breadth expansion (which complements the breadth expansion on the array *a*) uses the unwind expansion operator (ueo) to apply the result of the nested depth expansion on all elements found under *k* in *t*.

**Example: semantics** We now report excerpts of the execution of the unwind operator at Line 9 of Listing 2 to exemplify both the unfolding of the breadth and depth expansions.

We remind that — sl— has the shape reported in Line 7 in Listing 1 and that the application at Line 9 of Listing 2 “unwinds” the — sl— data structure with path $\underline{M.D.L}$.

The first expansion we perform is the breadth expansion over the array — sl—. Since — sl— just contains one tree, *i.e.,* that for sleep logs of 2020, we just have one application of the ueo operator (the empty array  [] at the right of the concatenation operator :: results from the “otherwise” branch of the definition of the unwind and from— sl— being structurally equivalent to *sl*[1]:: []).

 
 
 
   _______________________________________________________________________________________ 
 
   ueo (sl [1], ω([[ M.ɛ  ]]sl [1] , D.L  ), M) :: [ ] 
    

Then, we show the “depth” part of the expansion, by focusing on the terminal part of the application of the ueo operator. Specifically, we concentrate on the tree corresponding to the sleep logs of day 2020-11-29, found at Line 11 of Listing 1 and aliased with the tree *t*_29_. Formally, the expansion corresponds to the application $\mathsf{ueo}({t}_{29},[ [\underline{L.}] ]^{{t}_{29}},L)$ of the terminal node *L* in path $\underline{M.D.L}$.

 
 
 
  _______________________________________________________________________________________________________________________________________ 
 
  ueo ( t29 , [[ L.ɛ]] t29 , L ) ⇒ 
   [ ( ν { d : [ 29 { } ] , L : [ ... ] } \ ν { L : [ ... ] } ) ∪ ν { L : [ ν { s : [ ' 21:01 ' { } ] , e : [ ' 03:12 ' 
    { } ] , q : [ ' good ' { } ] } ] } ] :: 
    [ ( ν { d : [ 29 { } ] , L : [ ... ] } \ ν { L : [ ... ] } ) ∪ ν { L : [ ν { s : [ ' 03:36 ' { } ] , e : [ ' 09:58 ' 
    { } ] , q : [ ' good ' { } ] } ] } ]    

Above, for each element of the array pointed by *L*, we create a new structure where we replace the original array associated with the key *L* with a new array containing only one element. For instance, the first element of the result takes the original structure found under *D* ( [*ν*  {*d*:[29{}], *L*: [... ] } ]) and updates it to contain only the element  
 
   υ { s : [ ' 21:01 ' { } ] , e : [ ' 03:12 ' { } ] , q : [ ' good ' { } ] }  associated to the node *L*.

#### The project operator

The purpose of the *project* operator is to modify the trees in an array *a* by projecting nodes, renaming node labels, or introducing new nodes, as described in the sequence of elements Π, which are either a path *p* or an injection (〈〉) of a *value definition d* into a path.

A value definition *d* can be (in the grammar, from left to right): (i) a value, (ii) a path, (iii) an array of value definitions, (iv) a criterion (*φ*) (cf. ‘The match operator”) or (v) a ternary expression on a criterion and two value definitions.

**Example** As done for the other operators, we draw our examples from Listing 2, where we have four usages of the project operator, the first at Line 5, the second at Line 10, the third at Line 13, and the fourth at Line 15. Here, we focus on the second example, at Line 10, reported in the snippet below. We comment on the others when exemplifying the *lookup* operator in ‘The lookup operator’.

 
 
 
  _________________________________________________________________________________________________________________________ 
 
  10 sl ← π ( sl , ( y  〈〉 year  , M.m  〈〉 month  , M.D.d  〈〉 day  , M.D.L.q  〈〉 quality 
) ) 
 
    

The projection at Line 10 takes the — sl— data structure resulting from the application of the unwind at Line 9 and performs a sequence of renaming over all tress within — sl—. For each tree, we perform the rename of the node *y* in *year* by moving the content of path $\underline{y}$ into the node corresponding to path $\underline{year}$, represented by the fragment $\underline{y}\langle \rangle \underline{year}$. Similarly, we move the content of $\underline{M.m}$ under $\underline{month}$, of $\underline{M.D.d}$ under $\underline{day}$, and of $\underline{M.D.L.q}$ under $\underline{quality}$. The result of the projection is the following flattened structure:

 
 
 
  _________________________________________________________________________________________________________________________ 
 
  [ υ { year : [ 2020 { } ] , month : [ 11 { } ] , day : [ 27 { } ] , quality : [ ' 
 
   poor ' { } ] } , 
    υ { year : [ 2020 { } ] , month : [ 11 { } ] , day : [ 28 { } ] , quality : [ ' 
    good ' { } ] } , 
    υ { year : [ 2020 { } ] , month : [ 11 { } ] , day : [ 29 { } ] , quality : [ ' 
    good ' { } ] } , 
    ... ] 
    

**Semantics** We start by defining the auxiliary operators we use in the definition of the project. Auxiliary operators *π*(*a*, *p*) and *π*(*t*, *p*) formalise the application of a branch selection over a path *p* respectively over an array and a tree. Then, we define the auxiliary operator eval(*d*, *t*), which returns the array resulting from the evaluation of a value definition *d* over a tree *t*. Finally, we report the projection of an injection of a value definition *d* into a path *p* over a tree *t*, *i.e., π*(*t*, *d*〈〉*p*).

The projection *π*(*a*, *p*) for a path *p* over an array *a* results in an array whose elements are the projection for *p* of the elements of *a*:

\begin{eqnarray*}\pi (a,p)=\pi (~\text{[}{t}_{1},\ldots ,{t}_{n}~\text{]},p)=~\text{[}\pi ({t}_{1},p),\ldots ,\pi ({t}_{n},p)~\text{]} \end{eqnarray*}

The projection *π*(*t*, *p*) for a path *p* over a tree *t* implements the actual semantics of branch selection, where, given a path *e*.*p*′ with *e*↓*k*, we remove all the branches  but  and continue to apply the projection for the continuation *p*′ over the (array of) sub-trees under *k* in *t* (*i.e.,* $[ [\underline{k.}] ]^{t}$). Formally:

\begin{eqnarray*}\pi (t,p)= \left\{ \begin{array}{@{}ll@{}} \displaystyle \upsilon ~\text{\{}k:\pi ([ [\,\underline{k.\epsilon }\,] ]t,{p}^{{^{\prime}}})~\text{\}}&\displaystyle \text{if}[ [\,p\,] ]^{t}\not = \alpha \wedge p=e.{p}^{{^{\prime}}}\wedge e \downarrow  k\\ \displaystyle t &\displaystyle \text{if}p=\\ \displaystyle \tau  &\displaystyle \text{otherwise} \end{array} \right. \end{eqnarray*}

The operator eval(*d*, *t*) evaluates the value definition *d* over the tree *t* and returns an array containing the result of the evaluation. Formally:

\begin{eqnarray*}\mathsf{eval}(d,t)= \left\{ \begin{array}{@{}ll@{}} \displaystyle ~\text{[}d~\text{\{}\text{\}}~\text{]}&\displaystyle \text{if}d\in V\\ \displaystyle [ [\,d\,] ]^{t} &\displaystyle \text{if}d\in P\\ \displaystyle \mathsf{eval}(d,t)::\mathsf{eval}({d}^{{^{\prime}}},t) &\displaystyle \text{if}d=~\text{[}d~\text{]}::{d}^{{^{\prime}}}\\ \displaystyle ~\text{[}t\models \varphi ~\text{\{}\text{\}}~\text{]}&\displaystyle \text{if}d=\varphi \\ \displaystyle \mathsf{eval}({d}_{1},t) &\displaystyle \text{if}d=\varphi {?}{d}_{1}:{d}_{2}\wedge t\models \varphi \\ \displaystyle \mathsf{eval}({d}_{2},t) &\displaystyle \text{if}d=\varphi {?}{d}_{1}:{d}_{2}\wedge t\not \models \varphi \\ \displaystyle \alpha  &\displaystyle \text{otherwise} \end{array} \right. \end{eqnarray*}

The projection *π*(*t*, *d*〈〉*p*) of the injection of the evaluation of a value definition *d* on a tree *t* into a path *p* results in a new tree where we find the evaluation of *d* on *t* under *p*.

\begin{eqnarray*}\pi (t,d\;\langle \rangle \;p)= \left\{ \begin{array}{@{}ll@{}} \displaystyle \upsilon ~\text{\{}k:~\text{[}\pi (t,d\;\langle \rangle \;{p}^{{^{\prime}}})~\text{]}~\text{\}}&\displaystyle \text{if}p=e.{p}^{{^{\prime}}}\wedge e \downarrow  k\wedge \mathsf{eval}(d,t)\not = \alpha \\ \displaystyle \upsilon ~\text{\{}k:\text{eval}(d,t)~\text{\}} &\displaystyle \text{if}p=e.\wedge e \downarrow  k\wedge \mathsf{eval}(d,t)\not = \alpha \\ \displaystyle \tau  &\displaystyle \text{otherwise} \end{array} \right. \end{eqnarray*}

Before formalising the projection, we report the auxiliary operator ⊕ to merge arrays and trees—we use the operator to merge the result of sequences of projections in the definition of *π*(*t*, Π).

\begin{eqnarray*}\begin{array}{@{}c@{}} \displaystyle (~\text{[}t~\text{]}::a)\;\oplus \;(~\text{[}{t}^{{^{\prime}}}~\text{]}::{a}^{{^{\prime}}})=~\text{[}t\;\oplus \;{t}^{{^{\prime}}}~\text{]}::a\oplus {a}^{{^{\prime}}}\nonumber\\\displaystyle t\oplus \tau =t a\oplus ~\text{[}\text{]}=~\text{[}\text{]}\oplus a=a\oplus \alpha =\alpha \oplus a=a \end{array} \end{eqnarray*}

\begin{eqnarray*} \frac{t=b~\text{\{}{k}_{i}:{a}_{i}~{\text{\}}}_{i\in I}\wedge {t}^{{^{\prime}}}=b~\text{\{}{k}_{j}:{a}_{j}~{\text{\}}}_{j\in J}}{t\oplus {t}^{{^{\prime}}}=b~\text{\{}{k}_{h}:{k}_{h}(t)\oplus {k}_{h}({t}^{{^{\prime}}})~{\text{\}}}_{h\in I\cup J}}   \frac{b\not = {b}^{{^{\prime}}}}{b~\text{\{}{k}_{i}:{a}_{i}~{\text{\}}}_{i}\oplus {b}^{{^{\prime}}}~~\text{\{}{k}_{j}:{a}_{j}~{\text{\}}}_{j}=\tau } \end{eqnarray*}

To conclude, we first report the application of the projection to a tree *t*, *π*(*t*, Π), which merges the results of projections in Π over *t* into a single tree. Second, we report the application of the projection to an array *a*, *π*(*a*, Π), which corresponds to the application of the projection to all elements of *a*. Respectively, we formally write:

\begin{eqnarray*}\pi (t,\Pi )= \left\{ \begin{array}{@{}ll@{}} \displaystyle \pi (t,p)\oplus (\pi (t,{\Pi }^{{^{\prime}}})) &\displaystyle \text{if}\Pi =p,{\Pi }^{{^{\prime}}}\\ \displaystyle \pi (t,d\;\langle \rangle \;p)\oplus (\pi (t,{\Pi }^{{^{\prime}}})) &\displaystyle \text{if}\Pi =d\;\langle \rangle \;p,{\Pi }^{{^{\prime}}}\\ \displaystyle \pi (t,p) &\displaystyle \text{if}\Pi =p\\ \displaystyle \pi (t,d\;\langle \rangle \;p) &\displaystyle \text{if}\Pi =d\;\langle \rangle \;p \end{array} \right. \end{eqnarray*}

and

\begin{eqnarray*}\pi (a,\Pi )=\pi (~\text{[}{t}_{1},\ldots ,{t}_{n}~\text{]},\Pi )=~\text{[}\pi ({t}_{1},\Pi ),\ldots ,\pi ({t}_{n},\Pi )~\text{]} \pi (~\text{[}\text{]},\Pi )=\pi (\alpha ,\Pi )=~\text{[}\text{]} \end{eqnarray*}

**Example: semantics** We report the execution of the project at Line 10 of Listing 2. We take — sl— as returned after the application of the unwind operator described in ‘The unwind operator’. For brevity, we represent the — sl— data structure as the concatenation of its elements, *i.e., sl* = *sl*[1]::*sl*[2]::⋯.

 
 
 
   _______________________________________________________________________________________________________________________ 
 
   π ( sl [1] :: sl [2] :: ⋅⋅⋅ , 
 
   (y 〈〉 year  , M.m  〈〉 month  , M.D.d  〈〉 day  , M.D.L.q  〈〉 quality  )) ⇒ 
 
  [ π( sl [1],(y 〈〉 year  , M.m  〈〉 month  , M.D.d  〈〉 day  , M.D.L.q  〈〉 quality  )) , 
 
   π( sl [2],(y 〈〉 year  , M.m  〈〉 month  , M.D.d  〈〉 day  , M.D.L.q  〈〉 quality  )) , ... ]    

We continue showing the projection of the first element in *a*, *sl*[1] (the projection on the other elements follows the same structure)  
 
 
   _______________________________________________________________________________________________________________________ 
 
   π( sl [1],(y 〈〉 year  , M.m  〈〉 month  , M.D.d  〈〉 day  , M.D.L.q  〈〉 quality  )) ⇒ 
 π( sl [1],y 〈〉 year  ) ⊕ π( sl [1],M.m 〈〉 month  ) ⊕ π( sl [1],M.D.d 〈〉 day  ) ⊕ π( sl 
   [1],M.D.L.q 〈〉 quality  ) 


Finally, we show the unfolding of the first two projections from the left, above, *i.e.,* those for $\underline{y}\langle \rangle \underline{year}$ and for $\underline{M.m}\langle \rangle \underline{month}$, and their merge ⊕ (the remaining ones unfold similarly).


 
 
 
    π( sl [1],y 〈〉 year  ) ⊕ π( sl [1],M.m 〈〉 month  ) 
     ⇒ υ { year : π( sl [1],y ) } ⊕ υ { month : π( sl [1],M.m ) } 
   ⇒ υ { year : eval (y , sl [1]) } ⊕ υ { month : eval (M.m , sl [1]) } 
   ⇒ υ { year : [[ y ]]sl [1] } ⊕ υ { month : [[ M.m ]]sl [1] } 
   ⇒ υ { year : [ 2020 { } ] } ⊕ υ { month : [ 11 { } ] } 
   ⇒ υ { year : [ 2020 { } ] , month : [ 11 { } ] }    


#### The group operator


\begin{eqnarray*}\gamma (a,\Gamma ,{\Gamma }^{{^{\prime}}})\qquad \Gamma \,::=\,p\;\mid \;p\;\langle \rangle \;{p}^{{^{\prime}}}\;\mid \;p,\Gamma \;\mid \;p\;\langle \rangle \;{p}^{{^{\prime}}},\Gamma \end{eqnarray*}

The purpose of the *group* operator is to group the trees in an array *a* according to a specification Γ′ and to aggregate the values of the grouped trees according to the specification Γ. Both Γ and Γ′, respectively the *aggregation* and the *grouping* set, are sequences of elements of the form *p*〈〉*p*′ where *p* is a path in the input trees, and *p*′ a path in the output trees.

Note that Γ includes both fragments of the shape *p* and *p*〈〉*p*′. Here, the former is syntactic sugar for the latter, where both paths are the same. Therefore, we assume to apply the semantics of the group operator only with the de-sugared form *γ*(*a*, Γ, Γ′) = *γ*(*a*, exp(Γ), exp(Γ′)), where

\begin{eqnarray*}\mathsf{exp}({\Gamma }_{1},{\Gamma }_{2})=\mathsf{exp}({\Gamma }_{1}),\mathsf{exp}({\Gamma }_{2}) \mathsf{exp}(p)=p\;\langle \rangle \;p \mathsf{exp}(q\;\langle \rangle \;p)=q\;\langle \rangle \;p \end{eqnarray*}

**Example** Drawing from Listing 2, we have two applications of the group operator, one at Line 4 and the second at Line 12. Since the two applications are similar, we just focus on the latter (reported below), leaving the comment on the second to ‘The lookup operator’.

 
 
 
12 sl ← γ ( sl , ( quality  ) , () )    

As stated above, the aggregation set expands from $\underline{quality}$ to the de-sugared form $\underline{quality}\langle \rangle \underline{quality}$.

The group operator applies on the data structure in — sl— which, at Line 11, we filtered with the match operator to only contain values corresponding to the dates 2020-11-29 and 2020-11-30. The new data structure, copied into — sl— and reported below, is essentially the aggregation under the node *quality* of the filtered sleep recordings.

 
 
 
  _______________________________________________________________________________________________________________________ 
 
  [ υ { quality : [ ' good ' { } , ' good ' { } , ' poor ' { } , ' good ' { } ] } 
  ]    

To make for a more comprehensive illustration, in this section we consider an alternative version of the example above, where we want to use the group operator to group the values by $\underline{day}$, $\underline{month}$, and $\underline{year}$ and aggregate the values of the sleep $\underline{quality}$. Concretely, we do this by updating the command found at Line 12 with the sequence of paths replacing the third parameter, which in the original we left empty.

 
 
 
  _________________________________________________________________________________________ 
 
  sl ← γ ( sl , ( quality  ) , ( day  , month  , year  ) )    

As stated, the paths $\underline{quality}$, $\underline{day}$, $\underline{month}$, and $\underline{year}$ respectively expand to $\underline{quality}\langle \rangle \underline{quality}$, $\underline{day}\langle \rangle \underline{day}$, $\underline{month}\langle \rangle \underline{month}$, and $\underline{year}\langle \rangle \underline{year}$.

The main detail we want to notice here is that, by grouping the values by $\underline{year}$, $\underline{month}$, and $\underline{day}$, we only aggregate logs relative to the same day.

 
 
 
  ______________________________________________________________________________________________________________________ 
 
  [ υ { year : [ 2020 { } ] , month : [ 11 { } ] , day : [ 29 { } ] , quality : [  ' good  ' { } ,  ' 
     good  ' { } ] } , 
    υ { year : [ 2020 { } ] , month : [ 11 { } ] , day : [ 30 { } ] , quality : [  ' poor  ' { } ,  ' 
     good  ' { } ] } 
   ]    

**Semantics** We start by reminding the shape of the de-sugared syntax of the group operator.

\begin{eqnarray*}\gamma (a,\Gamma ,{\Gamma }^{{^{\prime}}})=\gamma (a,\mathsf{exp}(\Gamma ),\mathsf{exp}({\Gamma }^{{^{\prime}}}))=\gamma (a~,~{{q}_{1}\langle {p}_{1},\ldots ,{q}_{n}\rangle {p}_{n}}_{\text{aggregation set}~\mathcal{A}}~,~{{s}_{1}\langle {r}_{1},\ldots ,{s}_{m}\rangle {r}_{m}}_{\text{grouping set}~\mathcal{G}}) \end{eqnarray*}

Intuitively, the group operator performs the following actions:

(a) it groups together those trees in *a* that (1) have the maximal number of existing paths from the grouping set *s*_1_, …, *s*_*m*_ and (2) whose values under those paths coincide;
(b) it projects the values in the grouped trees from *s*_1_, …, *s*_*m*_ to the corresponding paths *r*_1_, …, *r*_*m*_;
(c) it aggregates all the values in the grouped trees found under the paths *q*_1_, …, *q*_*n*_ from the aggregation set;
(d) it projects the aggregated values from *q*_1_, …, *q*_*n*_ into the corresponding paths *p*_1_, …, *p*_*n*_.

Formally, let *S* = {*s*_1_, …, *s*_*m*_} be the set of left elements in the injections of the sequence in the grouping set and let Σ be the power-set 2^*S*^ of paths in *S* so that

\begin{eqnarray*}\Sigma = \left\{ \varnothing ,\{ {s}_{1}\} ,\{ {s}_{2}\} ,\{ {s}_{3}\} ,\ldots ,\{ {s}_{1},{s}_{2}\} ,\{ {s}_{1},{s}_{3}\} ,\ldots ,\{ {s}_{1},\ldots ,{s}_{m}\} \right\} =\{ {\sigma }_{1},\ldots ,{\sigma }_{k}\} \end{eqnarray*}

We define the auxiliary operator exists which takes *S* and an element *σ* ∈ Σ and builds the *existence-match-query* formula of the paths in *S* w.r.t. the combination identified by *σ*.

\begin{eqnarray*}\mathsf{exists}(\sigma ,S)= \left\{ \begin{array}{@{}ll@{}} \displaystyle \mathtt{true} &\displaystyle \text{if}S=\varnothing \\ \displaystyle \exists s\wedge \mathsf{exists}(\sigma ,S\setminus \{ s\} ) &\displaystyle \text{let}s\in S\text{and}s\in \sigma \\ \displaystyle \neg \exists s\wedge \mathsf{exists}(\sigma ,S\setminus \{ s\} ) &\displaystyle \text{let}s\in S\text{and}s\not \in \sigma \end{array} \right. \end{eqnarray*}

We use the exists operator to perform part 1) of Item (a), *i.e.,* grouping those trees in *a* so that the trees in the same group have the same set of existing and non-existing paths from *s*_1_, …, *s*_*m*_. The part operator (presented below) performs part 2) of Item (a), which is the partition of the trees grouped by the exists operator so that the values in their existing paths in *s*_1_, …, *s*_*m*_ coincide.

We now define the semantics of the group operator and then present the semantics of the part operator. In the remainder, to make the definitions more intuitive, we alias the aggregation set with $\mathcal{A}$ and the grouping set with $\mathcal{G}$. Let, *k* = |Σ|, we write

\begin{eqnarray*}\gamma (a,\mathcal{A},\mathcal{G})=\mathsf{part}(\mu (a,\mathsf{exists}({\sigma }_{1},S)),{\sigma }_{1},\mathcal{A},\mathcal{G})::\cdots ::\mathsf{part}(\mu (a,\mathsf{exists}({\sigma }_{k},S)),{\sigma }_{k},\mathcal{A},\mathcal{G}) \end{eqnarray*}

As mentioned, the part operator finds the elements of *a* which should be grouped together according to $\mathcal{G}$ (among those selected through *σ*). In the definition, we delegate the actual grouping to the other auxiliary operator group, which (as hinted in Item (b)) projects the partitioned values from *S* into the corresponding destination path *r*_1_, *r*_2_, … in $\mathcal{G}$. The group operator also performs the aggregation of the values found in *q*_1_, *q*_2_, … (Item (c)) and it projects them under the corresponding destination path *p*_1_, *p*_2_, … (Item (d)).

In the semantics of the part operator, we assume to extend the set difference ∖ to arrays, so that *a*∖*a*′ returns a copy of *a* without the elements found in *a*′ (preserving their relative order). We also assume to have a variant of the match operator *μ*^*id*^(*a*, *φ*) that, instead of returning the array of trees in *a* that match the criterion *φ*, it returns the array of their indexes in *a*.

\begin{eqnarray*}\mathsf{part}(a,\sigma ,\mathcal{A},\mathcal{G})= \left\{ \begin{array}{@{}ll@{}} \displaystyle a &\displaystyle \text{if}a=~\text{[}\text{]}\\ \displaystyle \mathsf{group}(a,\sigma ,\mathcal{A},\mathcal{G}) &\displaystyle \text{if}\sigma ={0}\\ \displaystyle  &\displaystyle \text{otherwise, let}\sigma =\{ {s}_{1},\ldots ,{s}_{i}\} ,\\ \displaystyle \mathsf{group}(~\text{[}a[j],\ldots ,a[k]~\text{]},\sigma ,\mathcal{A},\mathcal{G})\nonumber\\\displaystyle \quad ::\mathsf{part}(~\text{[}a[f],\ldots ,a[g]~\text{]},\sigma ,\mathcal{A},\mathcal{G}) &\displaystyle {\mu }^{id}(a,{\mathop{\bigwedge }\nolimits }_{j=1}^{i}{s}_{j}=[ [\,{s}_{j}\,] ]^{a[1]})=~\text{[}j,\ldots ,k~\text{]},\\ \displaystyle  &\displaystyle ~\text{[}f,\ldots ,g~\text{]}=~\text{[}1,\ldots ,\text{#}a~\text{]}\setminus ~\text{[}j,\ldots ,k~\text{]} \end{array} \right. \end{eqnarray*}

Finally, we report below the definition of the group operator. There, the last case is where we aggregate the values found in the array *a* following the paths in $\mathcal{A}$, and we combine them with the grouped values from $\mathcal{G}$ by using the project operator. The aggregation of the values in *a* is done by invoking the group operator on the second case. The second case applies when *σ* = ∅ (*i.e.,* when no path *S* is selected for grouping). The result of the application of the second case is an array containing one tree that combines the values of the array *a* following the paths in $\mathcal{A}$. To aggregate the values, we use the auxiliary tree variant of the project operator (*π*(*t*, Π), cf. ‘The project operator’) to project each value for a given path *q* into its corresponding path *p* in $\mathcal{A}$.

\begin{eqnarray*}\mathsf{group}(a,\sigma ,\mathcal{A},\mathcal{G})= \left\{ \begin{array}{@{}l@{}} \displaystyle a  & \text{if}a=~\text{[}\text{]}\\ \displaystyle ~\text{[}\pi (\tau ,\underbrace{{\eta }_{1}\langle {p}_{1},\ldots ,{\eta }_{n}\rangle {p}_{n}{}}_{aggregation})~\text{]}  & \text{if}\sigma ={0}, \text{let} \mathcal{A}={q}_{1}\langle {p}_{1},\ldots ,{q}_{n}\rangle {p}_{n},\\ \displaystyle   & {\eta }_{j}=\pi (a,{q}_{j}), j\in [1,n]\\ \displaystyle \pi ({a}^{{^{\prime}}},\underbrace{[ [\,{s}_{i}\,] ]a[1]\langle {r}_{i},\ldots ,[ [\,{s}_{j}\,] ]a[1]\rangle {r}_{j}{}}_{grouping})  & \text{otherwise, let}{a}^{{^{\prime}}}=\mathsf{group}(a,{0},\mathcal{A},\mathcal{G})\\ \displaystyle   & \mathcal{G}={s}_{1}\langle {r}_{1},\ldots ,{s}_{m}\rangle {r}_{m},\\ \displaystyle   & \sigma =\{ {s}_{i},\ldots ,{s}_{j}\} ,1\leq i\leq j\leq m \end{array} \right. \end{eqnarray*}

**Example: semantics** To illustrate the semantics of the group operator, we consider the alternative version of the code shown at Line 12 (and presented as a second example at the beginning of this section), where we want to aggregate for $\underline{quality}$ but we also want to keep those values grouped by $\underline{year}$, $\underline{month}$, and $\underline{day}$.

 
 
 
   ______________________________________________________________________________________________________ 
 
   sl ← γ ( sl , ( quality  ) , ( day  , month  , year  ) )    

In the semantics, the first thing we do is the de-sugaring of paths—namely $\underline{quality}$, $\underline{day}$, $\underline{month}$, and $\underline{year}$, which respectively expand to $\underline{quality}\langle \rangle \underline{quality}$, $\underline{day}\langle \rangle \underline{day}$, $\underline{month}\langle \rangle \underline{month}$, and $\underline{year}\langle \rangle \underline{year}$—and then we apply the de-sugared group operator on — sl— (which, we remind, contains only values corresponding to the dates 2020-11-29 and 2020-11-30, represented by the trees ${t}_{29}^{1},{t}_{29}^{2},\ldots $ below).


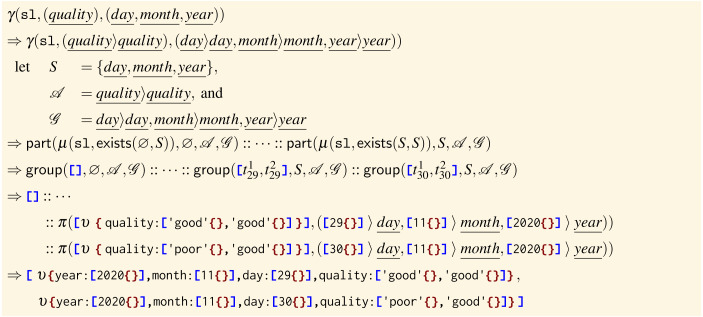


#### The lookup operator

*λ*(*a*, *q*, *a*′, *r*, *p*)

The purpose of the *lookup* operator is to join the trees in a source array *a* with the trees in an adjunct array *a*′. For those values obtained by applying the path *q* on *a*, the lookup operator pairs them with the equivalent values obtained by applying *r* on the adjunct array *a*′ and it projects the latter under path *p* in the paired trees of *a*.

**Example** Before commenting on the application of the lookup in Listing 2, we describe the results of the group at Line 4 and of the two projections, respectively at Line 5 and Line 13. At Line 4, we aggregate the temperatures in the — tmp— data structure, which results into


 
 
 
  __________________________________________________________________________________________________________ 
 
  [ υ { t : [ 36 { } , 36 { } , 37 { } ] } ]    


The projection at Line 5 performs two actions over the — tmp— data structure. First, it keeps only the node *t* (holding the temperatures filtered for the days of interest). Second, it projects into the filtered data structure the pseudo-identifier (— pseudoID—) under the node *patient*_*id*.

 
 
 
  ________________________________________________________________________________________________________________________ 
 
  [ υ { t : [ 36 { } , 36 { } , 37 { } ] , patient_id : [ ' id_xxx ' { } ] } ]    

The projection at Line 13, similar to the one above, keeps only the node *quality* (holding the quality of the sleep for the days of interest) and it projects the — pseudoID— under the node *patient*_*id*.

 
 
 
  _____________________________________________________________________________________________________________________ 
 
  [ υ { quality : [ ' good ' { } , ' good ' { } , ' poor ' { } , ' good ' { } ] , 
   patient_id : [ ' id_xxx ' { } ] } ]    

We can now comment on the lookup at Line 14, which we report below for convenience.

 
 
 
  _______________________________________________________________________ 
 
  14 bs ← λ ( sl , patient id  , tmp , patient id  , temperatures  )    

The instruction joins the data structures — tmp— and — sl— by pairing the values under the path $\underline{patient\text{_}id}$ (this is a special case where the left and right paths of the join coincide, *i.e.,* the path $\underline{patient\text{_}id}$). The last path in the application, *i.e.,* $\underline{temperatures}$, indicates where the values from the right data structure (— tmp—) should be projected in the paired values of the left one (— sl—).

At Line 14, we store the result of the application of the lookup into a new variable — bs— (standing for bio-signals).

 
 
 
  ____________________________________________________________________________________________________________________________________ 
 
  υ { quality : [ ' good ' { } , ' good ' { } , ' poor ' { } , ' good ' { } ] , 
   temperatures : [ υ { t : [ 36 { } , 36 { } , 37 { } ] , 
   patient_id : [   ' id_xxx ' { } ] } ] , 
   patient_id : [   ' id_xxx ' { } ] }    

For completeness, we report the result of the last step of Listing 2, at Line 15, where we apply the project operator to reshape the data structure for the invocation of the — detectEncephalopathy— functionality at Line 16.

 
 
 
  _________________________________________________________________________________________________________________________ 
 
  υ { quality : [ ' good ' { } , ' good ' { } , ' poor ' { } , ' good ' { } ] , 
   temperatures : [ 36 { } , 36 { } , 37 { } ] , 
   patient_id : [   ' id_xxx ' { } ] }    

**Semantics** In the semantics of the lookup, for each element *a*[*i*] (1 ≤ *i* ≤ #*a*), we use the tree version of the project operator (*π*(*t*, Π), cf. ‘The project operator’) to merge the element *a*[*i*] with the paired values from *a*′ under *r*. Since, by its definition, *π*(*t*, Π) corresponds to the merging (⊕) of the single applications of each component in the sequence Π, we use this to merge the source tree *a*[*i*] with the paired elements in *a*′. Hence, for each element *a*[*i*], we define Π_*i*_ as the sequence *ɛ*, *μ*(*a*′, *φ*_*i*_)〈〉*p*. The projection for the first component (*ɛ*) returns the original tree (*a*[*i*]). The projection for the second component (*μ*(*a*′, *φ*_*i*_)〈〉*p*) injects the result of the match *μ*(*a*′, *φ*_*i*_) into the path *p*, where the criterion *φ*_*i*_, equal to *r* = [[*q*]]^*a*[*i*]^, selects those values in *a*′ that under *r* coincide with the array found under *q* in *a*[*i*].

Note that when for some *i* we have *q* not present in *a*[*i*] (*i.e.,* [[*q*]]^*a*[*i*]^ = *α*) the lookup operator joins *a*[*i*] with those trees in *a*′ where *r* does not exist (*i.e., μ*(*a*′, *r* = *α*)).

\begin{eqnarray*}\lambda (a,q,{a}^{{^{\prime}}},r,p)=~\text{[}\pi (a[1],{\Pi }_{1})~\text{]}::\cdots ::~\text{[}\pi (a[\mathtt{#}a],{\Pi }_{\mathtt{#}a})~\text{]} \text{where}  \left\{ \begin{array}{@{}l@{}} \displaystyle 1\leq i\leq \mathtt{#}a \\ \displaystyle {\Pi }_{i}=,\mu ({a}^{{^{\prime}}},{\varphi }_{i})\;\langle \rangle \;p \\ \displaystyle {\varphi }_{i}=(r=[ [\,q\,] ]^{a[i]})  \end{array} \right. \end{eqnarray*}

**Example: semantics** Below, we report the unfolding of the execution of the lookup at Line 14. Since we have one value in — sl—, we do not perform a concatenation of arrays but we just apply the projection for — sl—[1]. In the three reductions below, first, we retrieve the content of $[ [\underline{patient\text{_}id}] ]^{\mathtt{sl}[1]}$, then, we execute the match (which essentially returns the whole content of the — tmp— variable), and, finally, we merge — sl—[1] (obtained by the projection under *ɛ*) with the result of the match projected under path $\underline{temperatures}$.


 
 
 
  _________________________________________________________________________________________________________________________ 
 
  [ π( sl [1],(ɛ,μ( tmp ,patient id = [[ patient id ]]sl [1] ) 〈〉 temperatures  )) ] 
    ⇒ [ π( sl [1],(ɛ,μ( tmp ,patient id = [ ' id_xxx ' { } ] ) 〈〉 temperatures  )) ] 
    ⇒ [ π( sl [1],(ɛ, [ υ { t : [ 36 { } , 36 { } , 37 { } ] , patient_id : [ ' id_xxx ' { } ] } ] 
    〈〉 temperatures  )) ] 
    ⇒ [ υ { quality : [ ' good ' { } , ' good ' { } , ' poor ' { } , ' good ' { } ] , 
    patient_id : [ ' id_xxx ' { } ] } ⊕ υ { temperatures : [ υ { t : [ 36 { } , 36 { 
   } , 37 { } ] , 
    patient_id : [ ' id_xxx ' { } ] } ] } ]    


## Implementation

We now present Jolie/Tquery, our implementation of Tquery as a Jolie microservice. Specifically, we chose to release Jolie/Tquery as a library that users can include and invoke locally in their Jolie projects—as an npm package (https://www.npmjs.com/package/@jolie/tquery.). However, thanks to Jolie’s module system, users can also expose Jolie/Tquery as an independent service, *e.g.*, as a RESTful service (Montesi, 2016) as well as a publish/subscribe MQTT worker (Gabbrielli et al., 2018) (as briefly detailed in ‘The implementation of Jolie/Tquery’).

In this section, first, we describe the main components of Jolie/Tquery, specified through the abstractions provided by the Jolie language (which follow the typical partition of microservice components (Giallorenzo et al., 2021)), namely: its Application Programming Interfaces (API), its access points, and its logic/behaviour. In particular, APIs and access points^2^ 2These specify the network, transport, and application protocols, *e.g.*, HTTP/TCP/IP.describe how users interact with Jolie/Tquery, while the behaviour implements the semantics of Tquery (cf. ‘The Tquery Formalisation’).

Then, we slightly extend the API and behaviour of Jolie/Tquery to support query *pipelines*, *i.e.,* multi-stage queries where (*a*) the first stage uses the data provided as input, (*b*) each other stage transforms the data from the proceeding stage, and (*c*) the last stage returns its output back to the invoker. We have two main reasons for extending Jolie/Tquery with pipelines: (*i*) for efficiency, since it removes the overhead of data transmission between sequential stages (as, *e.g.*, in Listing 2 at Lines 3–5 and Lines 9–15); (*ii*) for familiarity with the MongoDB Aggregation Framework (MongoDB Inc., 2022), where users express queries as multi-stage transformations.

Finally, we show the implementation of the example from ‘Overview and Running Example’ in Jolie/Tquery, both using the original sequence of operators (cf. ‘Overview and Running Example’) and as a combination of multi-stage pipelines.

### The implementation of Jolie/Tquery

We start from the API of Jolie/Tquery and then present how Jolie allows us to provide the microservice as a library and to also have an efficient implementation of its engine.

**Figure 2:**
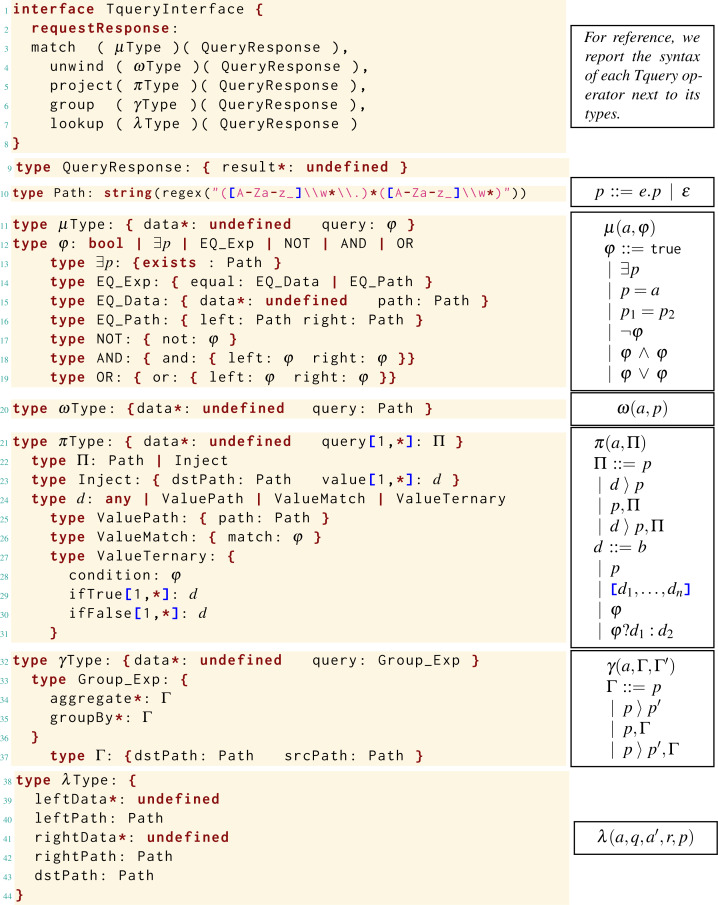
Mapping between the Tquery operators and Jolie/Tquery API.

**The Jolie/Tquery API** Simplifying (Giallorenzo et al., 2021; Montesi, Guidi & Zavattaro, 2014), in Jolie, the API of a microservice corresponds to an — interface—, which is a named collection of resources, called operations, each defined by a name, an interaction modality—i.e., asynchronous invocations or synchronous request responses (W3c, 2001)—and schemas of their expected inbound and outbound data, called — type—s. Thus, in Fig. 2, we report the API of Jolie/Tquery expressed as a Jolie — interface—, with its associated — type—s.

The code in Fig. 2 is a fragment of the main.ol ^3^ 3Available at https://github.com/jolie/tquery/blob/master/main.ol.executable Jolie file from Jolie/Tquery. In Fig. 2, we stylise the code omitting — void— root types (described in the following paragraph) and naming — type—s using the symbols from the formalisation. These conventions help keeping the code compact and also ease the comparison with Tquery, in unison with the boxed fragments reporting the Tquery syntax in Fig. 2.

We briefly introduce the main elements of Jolie APIs and we comment on the choices that drove the design of the Jolie/Tquery API. At Lines 1–8 of Fig. 2, we find the definition of — TqueryInterface—, the Jolie/Tquery — interface—. The keyword — requestResponse— indicates that the operations associated to it (as a comma-separated list) are synchronous invocations, where the caller waits for the callee (here, the Jolie/Tquery service) to reply with the computed response. We defined all the operations of Jolie/Tquery as — requestResponse—s since this interaction modality matches the invocation semantics of the Tquery operators.^4^ 4A possible alternative, here, is using asynchronous — oneWay—s and either choose a pull or push semantics to retrieve the results of the queries. We did not pursue this direction, since this modality would sensibly diverge from that of Tquery.

In the syntax of operations, *e.g.*,  
 
   match ( μ Type ) ( QueryResponse )     at Line 3, we find the name of the operation (  
 
   match ), the request — type— between the first parenthesis (  
Type ), and the response — type— between the second parenthesis (  
QueryResponse ).

A Jolie — type— has a name, *e.g.*,  
QueryResponse     at Line 9, and a shape similar to that of the trees described in ‘The Tquery Formalisation’: a root that contains a value (*e.g.*, — bool—, — int—, — string—, as well as the empty value, — void—) and sub-nodes that point to quantified arrays of typed trees, *e.g.*, the  
QueryResponse  — type— has a — void— root (omitted) and a sub-node named — result— which points to an unbounded array (— *—) of elements that can assume any shape (— undefined—).

Jolie — type—s can be further refined, *e.g.*, at Line 10, we restrict the set of strings that the root of the — type— — Path— can assume to those matching the regular expression within the — regex— predicate, following the definition of paths from Tquery.

Jolie — type—s support sum types (Pierce, 2002, Chapter 11) (Safina et al., 2016) of the shape — type— Name: LeftType—RightType. Here, we use sum types to keep the syntax of Tquery and the structure of Jolie/Tquery — type—s close. For example, at Line 12, we specify that the — type— *φ* can either be a — bool—ean, the — type— ∃*p*, etc..

**The Jolie/Tquery access points and behaviour** We now move to the description of the access points and the behaviour of Jolie/Tquery, reported in Listing 3. In Jolie, a microservice is identified by the keyword — service— associated with a name (in Listing 3, Tquery), a set of access points (in Listing 3, the — inputPort— at Lines 46–49), and a set of behaviours (in Listing 3, defined through the — foreign— language (— java—) at Lines 51–53).


 
 
 
  _________________________________________________________________________________ 
 
  45 service  Tquery  { 
   46  inputPort  IP  { 
   47  location : " local " 
   48  interfaces : TqueryInterface 
   49  } 
   50 
      51  foreign  java  { 
   52  class : " joliex . tquery . engine . TqueryService " 
   53  } 
   54 } 
               Listing 3: The Tquery service.    


Concerning access points, Jolie provides — inputPort—s to specify ingress gates, which define how a service expects clients to invoke its operations, and — outputPort—s (absent in Listing 3), which specify outbound egress gates for invoking other services (Montesi, Guidi & Zavattaro, 2014). At Line 46 of Listing 3, we define an — inputPort— (its name is immaterial here) with — location— — ”local”— and — interfaces— — TqueryInterface— (cf. Fig. 2). By specifying an inbound access point with a — ”local”— — location—, we indicate that our service accepts in-memory invocations from another Jolie service that runs Jolie/Tquery as an internal library—through a mechanism called “embedding” (Montesi, Guidi & Zavattaro, 2014).^5^ 5Jolie access points simplify the definition of alternative service configurations. For instance, to expose Jolie/Tquery as a RESTful service, we need to add a new — inputPort— (or change the one already defined) setting its — location— to a socket address (*e.g.*, — ”socket://localhost:8080”—) and its — protocol— to — http— (Montesi, 2016). In general, — protocol—s in Jolie specify the mapping between protocol-specific resources and Jolie operations and their data serialisation. Since the — ”local”— — location— transfers in-memory data structures, the definition of a — protocol— is unnecessary.

Regarding behaviours, Jolie provides a high-level language (akin to process calculi (Montesi, Guidi & Zavattaro, 2014)) to specify the composition of sophisticated workflows (Gabbrielli, Giallorenzo & Montesi, 2014) through a clean and minimal syntax. Jolie also supports the specification of behaviours through lower-level languages, like Java and Javascript, which are useful when integrating/exposing existing libraries as services or to manage lower-level abstractions like threads and pointers for performance. Jolie/Tquery falls in the latter category and we implemented its behaviour (and, thus, the Tquery semantics) using Java. This is visible at Lines 51–53 of Listing 3, where we declare the usage of the — foreign— language — java— to specify the — service— behaviour (implemented within the — TqueryService— class under the class-path — joliex.tquery.engine—). We omit the presentation of the Java code, since it closely follows the logic presented in ‘The Tquery Formalisation’.

#### Extending Jolie/Tquery with query pipelines

Besides providing a faithful implementation of Tquery, we decided to extend Jolie/Tquery to support multi-stage queries both for reasons of performance and familiarity with the MongoDB Aggregation framework (MongoDB Inc., 2022).

The extension is minimal and provides an interesting point for showcasing the flexibility of the Jolie language in evolving existing projects.

Namely, the extension regards the API and the behaviour. We report in Listing 5 the changes to the Jolie/Tquery API and we omit, as done above, to present the Java code of the implementation, which is a straightforward sequentialisation of calls to the other implemented operators.

In the API, we add the — pipeline— operation among the operations in the — TqueryInterface— — interface—. The new operation requires an associated request — type— that contains the specification of the multi-stage queries. Having defined the — type—s of the other operations as independent components comes in handy. Indeed, the — Pipeline— — type— defines its multi-stage query as an array (under the sub-node — pipeline—) of subtrees specified through the — type—s of the other operations. For instance, at Line 11 in Listing 5, a — match— (— Query—) stage has the structure of the *φ* — type—, which is also the one used by the — match— operation (in the  
Type     — type—). Here, the only exception is the — type— , which we did not use for the node — lookupQuery—, since the — leftData— sub-node is absent as the pipeline provides the (left-side) data.

 
 
 
   _____________________________________________________________________________ 
 
   1 interface  TqueryInterface  { 
   2  RequestResponse : 
  3  match  ( μ Type  ) ( QueryResponse  ) , 
  4   // ... 
  5  pipeline ( Pipeline  ) ( QueryResponse  ) 
  6 } 
   7 
      8 type  Pipeline : { 
   9  data * : undefined 
  10  pipeline [ 1 , * ] : 
  11  { matchQuery  : φ } 
   12   |  { projectQuery [ 1 , * ] : Π } 
   13   |  { unwindQuery  : Path  } 
   14   |  { groupQuery  : Group_Exp  } 
   15   |  { lookupQuery  : { 
   16  leftPath  : Path 
  17  rightData * : undefined 
  18  rightPath  : Path 
  19  dstPath  : Path 
  20  } 
   21  } 
   22 } 
         Listing 4: Pipeline support extension (fragments).    

The curious reader could wonder why we did not specify the whole Jolie/Tquery interface through the single — pipeline— operation. Our point is that, by having both possibilities, users can opt for the modality that best suits their scenario. For instance, when developing and debugging a query, it is useful to look at the shape of the single invocations and responses. Moreover, while pipelines help to make local sequential invocations efficient, they make the code harder to distribute, since the query now lives as an indivisible data structure. On the contrary, if we found out that a specific stage of a query, *e.g.*, the match at Line 3 or the unwind at Line 9 of Listing 2, would benefit from scaling it over multiple copies, we could do that by isolating each operation into a dedicated service and redirecting their inputs/outputs to perform our original local query as a distributed one. In that case, despite the architectural change, the logic of the query would remain intact.

### The Running Example written in Jolie/Tquery

**Figure 3:**
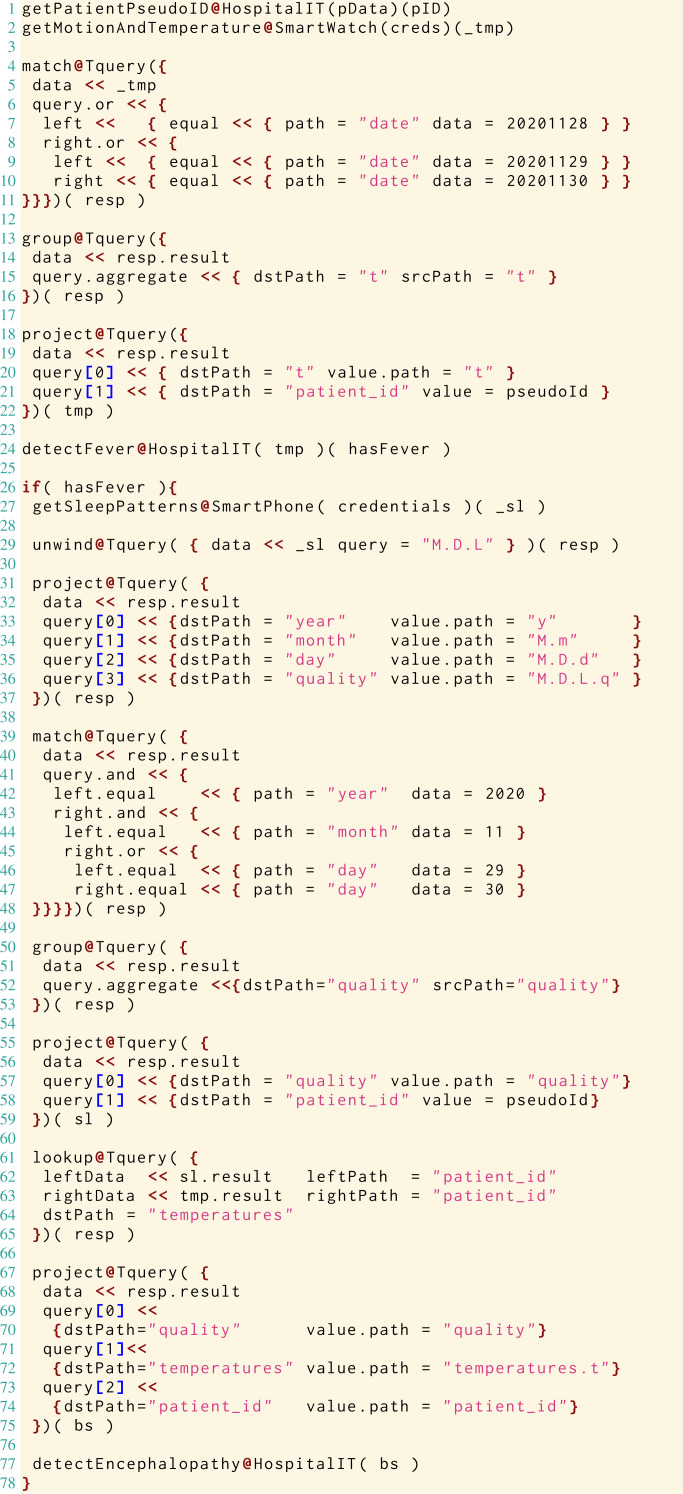
Single-stage implementation of Listing 2.

**Figure 4:**
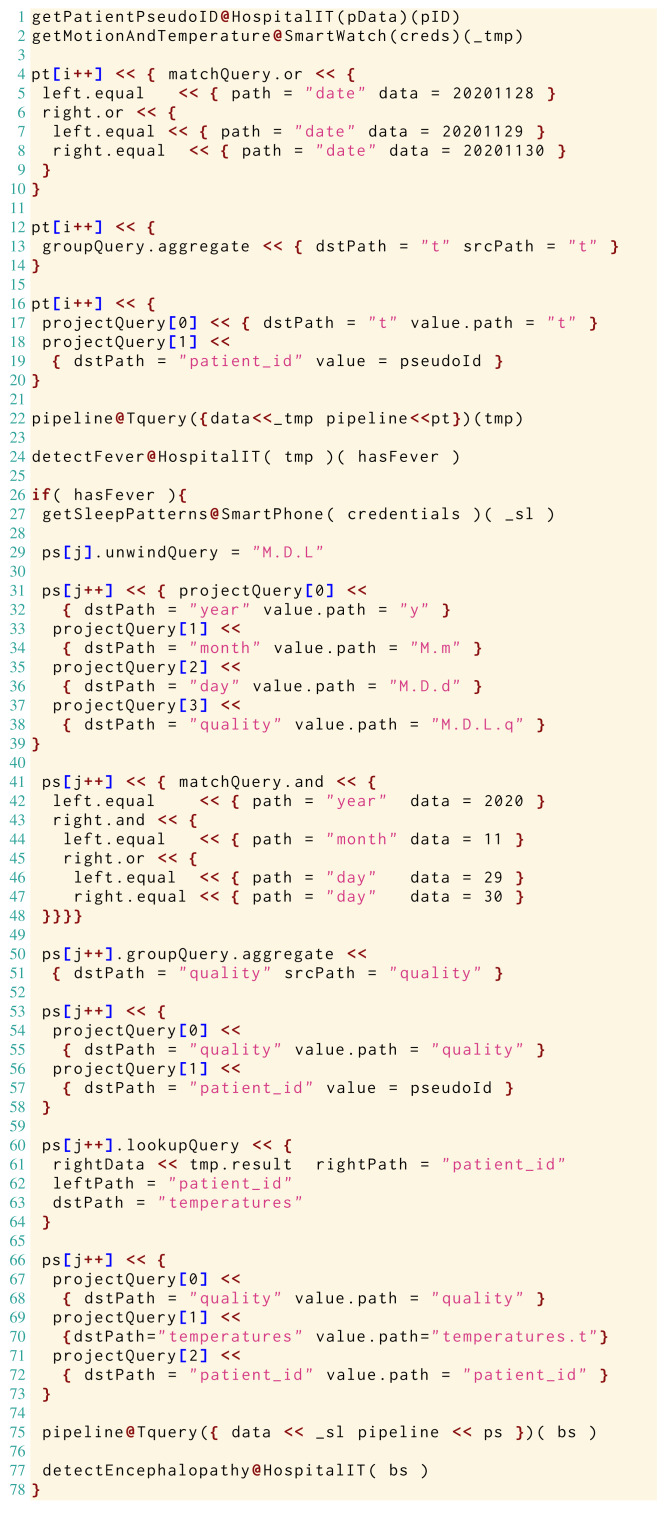
Multi-stage implementation of Listing 2.

We conclude this section by presenting the implementation of our running example from ‘Overview and Running Example’, Listing 2. Specifically, we present two alternatives: a more faithful one in Fig. 3, where we have a one-to-one correspondence between Tquery operators and Jolie/Tquery operations, and one in Fig. 4 that obtains the same result by using Jolie/Tquery pipelines.

While the code in Fig. 3 fulfills the promise made in ‘Overview and Running Example’ to show the implementation of the example in Listing 2, we take the chance to illustrate, in Fig. 4, how one can transition between a composition of single-stage queries to multi-stage, pipelined ones. Moreover, Fig. 4 is a reference for the actual Jolie/Tquery code used in ‘Benchmarks’ to benchmark our implementation.

Translating Tquery operator calls into Jolie/Tquery ones is straightforward, *e.g.*, the match at Line 3 of Listing 2 corresponds to Lines 4–11 of Fig. 3. As expected, the main difference is that we need to map the elements of the criterion *φ* from Line 3 of Listing 2 into a Jolie tree that follows the shape of type *φ* (cf. Lines 11–19 of Fig. 2).

The reuse of the — type—s of the single-stage operators in the definition of the — pipeline— helps migrating between the two modalities. For example, at Lines 4–10 of Fig. 4, we find that the definition of the match stage under the — pt— data structure follows the one at Lines 4–11 of Fig. 3.

We finally show how our implementation can interact with different services and heterogeneous data sources. In particular, we assume that the service offered by the hospital communicates XML messages over HTTP, and that smart-watches instead use an efficient binary protocol—SODEP (Montesi, Guidi & Zavattaro, 2014). These assumptions are coded in Jolie for our example with appropriate — outputPort—s that allow our implementation to contact these other components by using the right transports and data formats, as follows (we parameterise our code on the locations of these components, which are provided externally).

 
 
 
   _______________________________________________________________________________________________________________________ 
 
   1 outputPort  HospitalIT  { 
2  location : params . hospitalLocation 
 3  protocol : http  { format  = " xml "  } 
4  interfaces : HospitalInterface 
 5 } 
6 
7 outputPort  SmartWatch  { 
8  location : params . smartWatchLocation 
   9  protocol : sodep 
 10  interfaces : SmartWatchInterface 
   11 } 
        Listing 5: Collecting data from heterogeneous sources.    

The rest of our implementation is modular to these details: changing locations, protocols, or data formats does not require changing the code shown in Figs. 3 and 4.

## Benchmarks

We now present the method we followed to benchmark Jolie/Tquery and our experimental results. Specifically, we concentrate on the main application scenario of Tquery, *i.e.,* that of ephemeral data-handling, exemplified in ‘Overview and Running Example’ with the query logic presented in Listing 2. In ‘The Running Example written in Jolie/Tquery’ we showed two possible concrete realisations of the logic in Listing 2, developed using Jolie/Tquery. Here, we use Listing 2 as use case for our benchmarks and, as motivated below, the pipeline Jolie/Tquery realisation of Listing 2 (from Fig. 4), as the reference implementation to run our experiments.

To obtain a baseline against which to contrast the performance of Jolie/Tquery, we develop an alternative implementation of the example at ‘Overview and Running Example’ that uses MongoDB. This alternative implementation is the closest we can obtain to the logic expressed in ‘Overview and Running Example’, since *i*) the MongoDB query language (MongoDB Inc., 2022) inspired (*via* (Botoeva et al., 2018)) the design of Tquery and *ii*) the former supports a superset of the operators of the latter. As a confirmation of this fact, we implemented the logic of Listing 2 using MongoDB as a sequence of two, multi-stage queries, issued through the “aggregate” MongoDB API (https://docs.mongodb.com/manual/aggregation/). The resulting implementation follows the same invocation pattern as the one presented in ‘The Running Example written in Jolie/Tquery’, which uses the pipeline API extension of Jolie/Tquery, thus, motivating our choice to use this variant.

We remark that MongoDB provides an “in-memory” modality that avoids the overhead of making the data persistent on disk. Using this modality would likely give us baseline values closer to the in-memory performance profile of Tquery. Unfortunately, this modality is accessible only through the paid MongoDB Enterprise Advanced Subscription. Since using a paid-only feature would hinder the reproducibility of our experiments, we do not consider it. Here, we consider three configurations for MongoDB. First, the default one, tailored for persistency, that writes logs of transactions and data on disk. The second one is the MongoDB in the “no journal” modality, which avoids to write a log of the transactions on disk. The third one is an ephemeral configuration taken from grey literature (Girbal, 2021) that combines the “no journal” modality with the usage of a tmpfs (Snyder, 1990) disk as the one where MongoDB stores its data, to avoid the latencies of writing on non-volatile storage.

Below, we report the respective performance of the four configurations—one for Jolie/Tquery and three for MongoDB—in terms of the delay between when the engine receives a request and when it is ready to send back the response. Hence, we avoid recording the time spent transmitting the data between the invoker and the data-handling engine, which is orthogonal to the engine’s performance.

To run our benchmarks, we developed two Jolie microservices: one, called TqueryService, which contains the implementation in Fig. 4 and the other, called MongoService, which implements the following behaviour: (i) insert the data in MongoDB, (ii) perform the queries through MongoDB, and (iii) drop the data from MongoDB, to ensure ephemerality. When recording the performance of MongoService, we include the deletion (drop) time, before issuing back the response. To let MongoService and MongoDB interact, we use the synchronous version of the MongoDB Java Drivers^6^ 6Through the jolie-mongodb-driver library, available at https://github.com/szingaro/jmdb, which uses the MongoDB synchronous Java library, available at https://docs.mongodb.com/drivers/java/sync/current/.and we implement its behaviour in Java, similarly as done in ‘The implementation of Jolie/Tquery’ for Jolie/Tquery.

We synthetically generate 5 tiers of data for the benchmarks. Specifically, we generate 5 pairs of JSON files, each including one file for the temperatures and one for the sleep logs, following the structures from Listing 1. Each tier covers one year of recordings and it includes a number of samplings per day that doubles from a tier to the next: for the temperatures, the first tier contains one sampling per minute (1440 samplings per day), the second contains two samplings per minute (2880), and so on; for the sleep logs, the first tier contains 16 samplings per day, the second contains 32, and so on.

Our benchmark architecture includes a third Jolie microservice, called DataLoader, which we use to implement the high-level benchmark logic reported in Algorithm 1. Essentially, given the number of invocations to perform (*min*_*total*_*calls*), the number of requests in a batch (*batch*_*size*), and the set of data tiers (*tiers*), the service sends a sequence of *min*_*total*_*calls*/*batch*_*size* batches (rounded up to the next largest integer, to make sure to issue at least *min*_*total*_*calls* invocations). In Algorithm 1, the call *invokeTargetService* performs, in parallel, as many queries as indicated by the *batch*_*size*, where “*Target*” is one of the four configurations of our benchmark.

 
_______________________________________________________________________________________________________ 
 
  Algorithm 1: The DataLoader service logic. 
    Input: min_total_calls, batch_size, tiers 
    begin 
        for tier in tiers do 
       for batch ← 0 to ceil( min_total_calls / batch_size ) do 
   invokeTargetService ( tier, batch_size ) 
   end 
   end 
    end    

We execute our benchmarks on a machine equipped with an Intel Xeon Silver 4208 CPU @ 2.10 GHz (32 CPUs), 96GB RAM, and a Dell FH49G SSD. The machine runs CentOS 7 (Kernel 3.10.0 x86_64), Java 11 (with maximal heap size of 32GB), Jolie 1.10.5, Jolie/Tquery 0.4.10, the MongoDB Synchronous Driver 4.2.3, and MongoDB Community Server 4.4.6.

**Figure 5:**
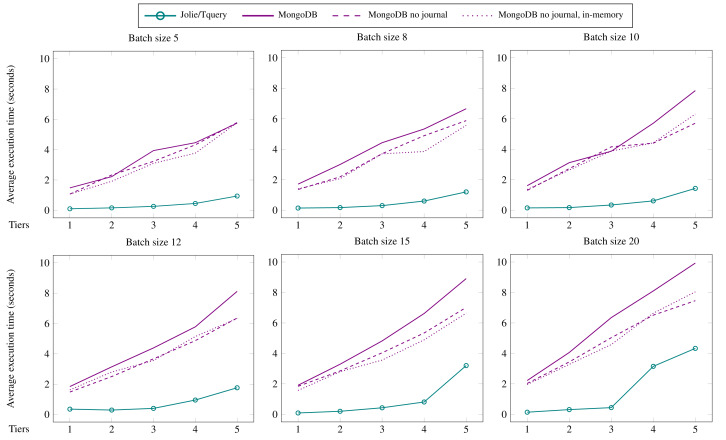
Batch-wise benchmarks for Jolie/Tquery and MongoDB.

We report in Fig. 5 our benchmarks of Jolie/Tquery and MongoDB, aggregated per batch size (from the top-left corner, for 5, 8, 10, 12, 15, and 20 parallel requests): each plot represents the relation between the data-tier size and the average execution time, maintaining constant the number of parallel invocations. The experimental results show that Jolie/Tquery performs consistently faster than MongoDB (all configurations). Since in the test cases with MongoDB we record the request-to-response delay of the database, the higher execution times of these cases correspond to both the overhead of the communication and the possible bottlenecks due to establishing connections to it. We notice a slight decrease in the relative distance between Jolie/Tquery and MongoDB at the increase of batch and data-tier sizes (in particular, the fourth and fifth tiers and the 15- and 20-sized batches). Our intuition of the phenomenon is that, on the given machine, when exceeding those thresholds, the Jolie execution runtime and the Jolie/Tquery engine undergo overhead due to resource contention. As expected, the default configuration of MongoDB is the one that performs the worst. The other two configurations (“no journal” and “no journal in-memory”) perform slightly better than the default and the difference between them is negligible—our intuition is that writing on disk is the driving factor that determines the drop in performance.

**Figure 6:**
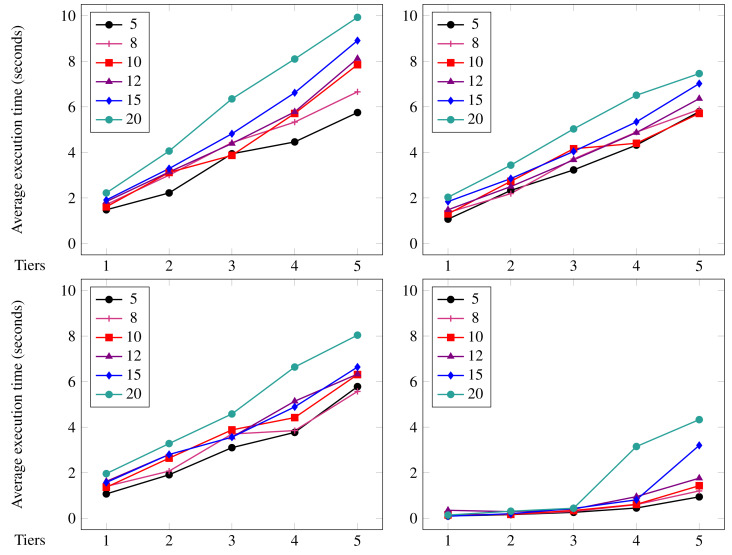
Engine-wise benchmarks of MongoDB (top-left), MongoDB without journaling (top-right), MongoDB without journaling and in-memory (bottom-left), and Jolie/Tquery (bottom-right). The lines represent the different batches of requests.

For completeness, we report in Fig. 6 the benchmarks aggregated by engine, which confirm the observations above: Jolie/Tquery consistently outperforms MongoDB over the different batches, where the degree of parallelism and the size of data are the main factors that determine changes in the performance trend.

Besides the direct results commented above, the performance behaviour plotted in Fig. 5 and Fig. 6 indicate that, when reaching some empirical threshold values, the system would benefit from scaling-up, either by distributing the query over multiple nodes or by having multiple copies of the same service and balancing the requests. Here, the flexibility of Jolie/Tquery can help the user to attain those configurations by minimising the footprint of the migration on both the system (no need to deploy additional database instances) and the codebase (cf. ‘Extending Jolie/Tquery with query pipelines’).

## Discussion and Conclusion

In this article, we presented Tquery, which is a theory for querying semi-structured data, compatible with Jolie. While Tquery is a formal model for general reference, we also presented Jolie/Tquery, which we showed to be especially suitable in the context of ephemeral data-handling. However, Jolie/Tquery is useful in general, for example in big-data analytics scenarios, where developers can specifies queries in a single node and then easily distribute it over different nodes.

Looking at future extensions, a natural evolution of this work is to perform a more complete evaluation of the expressivity of Jolie/Tquery by implementing well-known data-flow patterns (Hohpe & Woolf, 2004). A useful by-product of that endevour is the collection of a library of data-flow patterns implemented in Jolie/Tquery, available to developers. A complementary contribution to the above proposal is to perform an exhaustive study and benchmarking of the technologies for ephemeral data-handling. In that work, we would start by collecting real-world use cases of ephemeral data-handling and by selecting the most representative ones into a library of test scenarios. Then, we would collect the main tools used in ephemeral data-handling contexts (including Jolie/Tquery) and compare them from the different points of view of the features they have and their efficiency (*e.g.*, in terms of program size) and performance as obtained through the implementation of our library of tests.

Another direction is widening the scope of application of Jolie/Tquery with case studies and experiments where data queries are performed by low-power devices in IoT environments. This would entail building topologies of nodes with different tasks—e.g., gatherers (*e.g.*, edge devices equipped with sensors), collectors (*e.g.*, fog nodes that use Jolie/Tquery to aggregate and forward the gathered data to more powerful nodes), and crunchers (*e.g.*, cloud nodes where Jolie/Tquery would manage the high amount of data coming from the edge and fog layers)—and benchmarking their performance (possibly in comparison with alternative technologies for ephemeral data handling). Querying data on devices with low power and memory would likely require implementing strategies for distributing Jolie/Tquery pipelines over networks; future work in this direction will be able to benefit from the native support for services in heterogeneous environments offered by Jolie, which was another reason for developing a querying framework for Jolie.

We think that the above studies, besides providing us with the necessary material to guide us in evolving Jolie/Tquery—e.g., indicating the need for the inclusion of new operators—, would generate useful references for researchers to orient themselves in the growing field of ephemeral data-handling.

While studying the Tquery operators, we noticed and reported on how the shape of the data impacts on the possibility to distribute the stages of the query pipeline. To the best of our knowledge, this is a design space that did not receive a lot of attention in the literature and, yet, we deem it fundamental to provide further means for improving the performance of ephemeral data-handling systems. Here, our intuition is that Jolie types can help in providing a model that we can use to reason on the shape of the data and their interplay with the operators in a given query. Possible outcomes of this study include giving guidelines to developers to maximise the flexibility of their queries, as well as implementing tools that automatise the optimal distribution of query pipelines.

Finally, since Jolie/Tquery come as a library for the Jolie language, by implementing the support for new data formats in Jolie we would make them automatically available for Jolie/Tquery users.

##  Supplemental Information

10.7717/peerj-cs.1037/supp-1 Supplemental Information 1 Raw benchmarking data The JSON files that we generated to benchmark our system and the other scripts used for benchmarking.

## Additional Information and Declarations

### Data Availability

The following information was supplied regarding data availability: The code (test data is included in the tests directory) is available at GitHub: https://github.com/jolie/tquery.

## References

[ref-1] Apache (2005). Apache CouchDB. https://couchdb.apache.org.

[ref-2] Apache (2022a). Apache Flink. https://flink.apache.org.

[ref-3] Apache (2022b). Apache Samza. https://samza.apache.org.

[ref-4] Apache (2022c). Apache Storm. https://storm.apache.org.

[ref-5] Arasu A, Babcock B, Babu S, Cieslewicz J, Datar M, Ito K, Motwani R, Srivastava U, Widom J, Garofalakis M, Gehrke J, Rastogi R (2016). STREAM: the stanford data stream management system. Data stream management: processing high-speed data streams.

[ref-6] Arasu A, Babu S, Widom J (2006). The CQL continuous query language: semantic foundations and query execution. The VLDB Journal.

[ref-7] Armbrust M, Fox A, Griffith R, Joseph AD, Katz R, Konwinski A, Lee G, Patterson D, Rabkin A, Stoica I (2010). A view of cloud computing. Communications of the ACM.

[ref-8] Babcock B, Babu S, Datar M, Motwani R, Widom J (2002). Models and issues in data stream systems.

[ref-9] Babu S, Widom J (2001). Continuous queries over data streams. SIGMOD Record.

[ref-10] Baker SB, Xiang W, Atkinson I (2017). Internet of Things for smart Healthcare: technologies, challenges, and opportunities. IEEE Access.

[ref-11] Barbieri DF, Braga D, Ceri S, Della Valle E, Grossniklaus M (2009). C-SPARQL: sPARQL for continuous querying.

[ref-12] Botoeva E, Calvanese D, Cogrel B, Rezk M, Xiao G (2016). A formal presentation of MongoDB (Extended Version), CoRR.

[ref-13] Botoeva E, Calvanese D, Cogrel B, Xiao G, Kimelfeld B, Amsterdamer Y (2018). Expressivity and complexity of MongoDB queries.

[ref-14] Bray T, Paoli J, Sperberg-McQueen CM, Maler E, Yergeau F (2000).

[ref-15] Brian Krebs (2017). Extortionists wipe thousands of databases, victims who pay up get stiffed. https://krebsonsecurity.com/2017/01/extortionists-wipe-thousands-of-databases-victims-who-pay-up-get-stiffed.

[ref-16] Bunn JA, Navalta JW, Fountaine CJ, Reece JD (2018). Current state of commercial wearable technology in physical activity monitoring 2015-2017. International Journal of Exercise Science.

[ref-17] Callegati F, Gabbrielli M, Giallorenzo S., Melis A, Prandini M (2017). Smart mobility for all: a global federated market for mobility-as-a-service operators.

[ref-18] Caspi P, Pilaud D, Halbwachs N, Plaice JA (1987). LUSTRE: a declarative language for real-time programming.

[ref-19] Chen J, DeWitt DJ, Tian F, Wang Y (2000). NiagaraCQ: a scalable continuous query system for internet databases.

[ref-20] Cheney J, Lindley S, Wadler P (2013). A practical theory of language-integrated query. ACM SIGPLAN Notices.

[ref-21] Crockford D (2006). The application/json media type for javascript object notation (json). http://www.ietf.org/rfc/rfc4627.txt.

[ref-22] Diao Y, Fischer P, Franklin M, To R (2002). YFilter: efficient and scalable filtering of XML documents.

[ref-23] Dragoni N, Giallorenzo S, Lluch-Lafuente A, Mazzara M, Montesi F, Mustafin R, Safina L (2017). Microservices: yesterday, today, and tomorrow. Present and ulterior software engineering.

[ref-24] Elasticsearch (2022). Elasticsearch event query language. https://www.elastic.co/blog/introducing-event-query-language.

[ref-25] Ellis T (2014). Opaleye. https://github.com/tomjaguarpaw/haskell-opaleye.

[ref-26] Esteves S, Janssens N, Theeten B, Veiga L (2017). Empowering stream processing through edge clouds. SIGMOD Rec..

[ref-27] Fussel M (1997). Foundations of object-relational mapping. http://markfussell.emenar.com/blog/object-relational/.

[ref-28] Gabbrielli M, Giallorenzo S, Lanese I, Zingaro SP (2018). A language-based approach for interoperability of IoT platforms. https://scholarspace.manoa.hawaii.edu/server/api/core/bitstreams/573255ff-bc3a-4928-9f5b-3809a37745c3/content.

[ref-29] Gabbrielli M, Giallorenzo S, Lanese I, Zingaro SP, Majchrzak T, Mateos C, Poggi F, Grønli TM (2019). Linguistic abstractions for interoperability of IoT platforms. Towards integrated web, mobile, and IoT technology.

[ref-30] Gabbrielli M, Giallorenzo S, Montesi F, Omatu S, Bersini H, Corchado J, Rodríguez S, Pawlewski P, Bucciarelli E (2014). Service-oriented architectures: from design to production exploiting workflow patterns.

[ref-31] Giallorenzo S, Montesi F, Peressotti M, Rademacher F, Sachweh S, Damiani F, Dardha O (2021). Jolie and LEMMA: Model-Driven Engineering and Programming Languages Meet on Microservices.

[ref-32] Giallorenzo S, Montesi F, Safina L, Zingaro SP, Bertino E, Chang CK, Chen P, Damiani E, Goul M, Oyama K (2019). Ephemeral data handling in microservices.

[ref-33] Girbal A (2021). How to use MongoDB as a pure in-memory DB. https://edgystuff.tumblr.com/post/49304254688/how-to-use-mongodb-as-a-pure-in-memory-db-redis.

[ref-34] Hirten RP, Danieletto M, Tomalin L, Choi KH, Zweig M, Golden E, Kaur S, Helmus D, Biello A, Pyzik R (2020). Longitudinal physiological data from a wearable device identifies SARS-CoV-2 infection and symptoms and predicts COVID-19 diagnosis. MedRxiv.

[ref-35] Hirzel M, Schneider S, Gedik B (2017). SPL: an extensible language for distributed stream processing. ACM Transactions on Programming Languages and Systems.

[ref-36] Hohpe G, Woolf B (2004). Enterprise integration patterns: designing, building, and deploying messaging solutions.

[ref-37] Jang M (2006). Linux annoyances for geeks: getting the most flexible system in the world just the way you want it.

[ref-38] Kong L, Mamouras K (2020). StreamQL: a query language for processing streaming time series. Proceedings of the ACM on Programming Languages.

[ref-39] Leavitt N (2010). Will NoSQL databases live up to their promise?. Computer.

[ref-40] Ma M, Wang P, Chu C-H (2013). Data management for internet of things: challenges, approaches and opportunities.

[ref-41] Maschio B (2017). The use of microservices to implement cross process integration and data sharing. https://www.conf-micro.services/2017/papers/Maschio.pdf.

[ref-42] Maschio B (2019). Updating the current Jolie microservices based Document Management System to include electronic invoicing. https://www.conf-micro.services/2019/papers/Microservices_2019_paper_15.pdf.

[ref-43] Meijer E, Beckman B, Bierman G (2006). Linq: reconciling object, relations and xml in the. net framework. Sigmod.

[ref-44] Mendell M, Nasgaard H, Bouillet E, Hirzel M, Gedik B (2012). Extending a general-purpose streaming system for XML.

[ref-45] MongoDB Inc (2018a). MongoDB aggregation framework. https://www.mongodb.com/developer/products/mongodb/aggregation-framework/.

[ref-46] MongoDB Inc (2018b). MongoDB website. https://www.mongodb.com/.

[ref-47] MongoDB Inc (2022). Aggregation pipeline operators in MongoDB. https://docs.mongodb.com/manual/reference/operator/aggregation/.

[ref-48] Montesi F (2016). Process-aware web programming with Jolie. Science of Computer Programming.

[ref-49] Montesi F, Guidi C, Zavattaro G, Bouguettaya A, Sheng Q, Daniel F (2014). Service-oriented programming with Jolie. Web services foundations.

[ref-50] Mostert M, Bredenoord AL, Biesaart MC, Van Delden JJ (2016). Big Data in medical research and EU data protection law: challenges to the consent or anonymise approach. European Journal of Human Genetics.

[ref-51] Narkhede N (2017). Introducing KSQL: streaming SQL for Apache Kafka. https://www.confluent.io/blog/ksql-streaming-sql-for-apache-kafka/.

[ref-52] Oram A (2019). Ballerina: a language for network-distributed applications.

[ref-53] Pierce BC (2002). Types and programming languages.

[ref-54] Purohit B, Kumar A, Mahato K, Chandra P (2020). Smartphone-assisted personalized diagnostic devices and wearable sensors. Current Opinion in Biomedical Engineering.

[ref-55] Reda R, Piccinini F, Carbonaro A (2018). Towards consistent data representation in the IoT healthcare landscape.

[ref-56] Ron A, Shulman-Peleg A, Puzanov A (2016). Analysis and mitigation of NoSQL injections. IEEE Security & Privacy.

[ref-57] Rose N (2014). The human brain project: social and ethical challenges. Neuron.

[ref-58] Safina L, Mazzara M, Montesi F, Rivera V, Barolli L, Takizawa M, Enokido T, Jara AJ, Bocchi Y (2016). Data-driven workflows for microservices: genericity in Jolie.

[ref-59] Shein E (2013). Ephemeral Data. Communications of the ACM.

[ref-60] Shi W, Cao J, Zhang Q, Li Y, Xu L (2016). Edge computing: vision and challenges. IEEE Internet of Things Journal.

[ref-61] Siddhi (2022). Siddhi Streaming SQL. https://siddhi.io/en/v4.x/docs/query-guide/.

[ref-62] Snyder P (1990). tmpfs: a virtual memory file system.

[ref-63] Thurman SM, Wasylyshyn N, Roy H, Lieberman G, Garcia JO, Asturias A, Okafor GN, Elliott JC, Giesbrecht B, Grafton ST, Mednick SC, Vettel JM (2018). Individual differences in compliance and agreement for sleep logs and wrist actigraphy: a longitudinal study of naturalistic sleep in healthy adults. PLOS ONE.

[ref-64] Tommasini R, Sakr S, Balduini M, Valle ED (2019). An outlook to declarative languages for big steaming data.

[ref-65] Van Alsenoy B (2019). General data protection regulation. Data Protection Law in the EU: roles, responsibilities and liability.

[ref-66] Vigevano F, Liso PD (2018). Chapter 11 - differential diagnosis. Acute encephalopathy and encephalitis in infancy and its related disorders.

[ref-67] Visveswaran S (2000). Dive into connection pooling with J2EE. https://www.infoworld.com/article/2076221/dive-into-connection-pooling-with-j2ee.html.

[ref-68] W3c (2001). Transport message exchange pattern: single-Request-Response. https://www.w3.org/2000/xp/Group/1/10/11/2001-10-11-SRR-Transport_MEP.

[ref-69] WSO2 (2022). WSO2 stream processor. https://wso2.com/integration/streaming-integrator/.

